# Automated serial rotation electron diffraction combined with cluster analysis: an efficient multi-crystal workflow for structure determination

**DOI:** 10.1107/S2052252519007681

**Published:** 2019-07-12

**Authors:** Bin Wang, Xiaodong Zou, Stef Smeets

**Affiliations:** a Department of Materials and Environmental Chemistry, Stockholm University, Stockholm 106 91, Sweden

**Keywords:** serial crystallography, automated data collection, hierarchical cluster analysis, structure determination, electron diffraction, microED

## Abstract

Serial rotation electron diffraction (SerialRED) has been developed as a fully automated technique for three-dimensional electron diffraction data collection that can run autonomously without human intervention. It builds on the previously established serial electron diffraction technique, in which submicrometre-sized crystals are detected using image processing algorithms. Continuous rotation electron diffraction (cRED) data are collected on each crystal while dynamically tracking the movement of the crystal during rotation using defocused diffraction patterns and applying a set of deflector changes. A typical data collection screens up to 500 crystals per hour, and cRED data are collected from suitable crystals. A data processing pipeline is developed to process the SerialRED data sets. Hierarchical cluster analysis is implemented to group and identify the different phases present in the sample and to find the best matching data sets to be merged for subsequent structure analysis. This method has been successfully applied to a series of zeolites and a beam-sensitive metal–organic framework sample to study its capability for structure determination and refinement. Two multi-phase samples were tested to show that the individual crystal phases can be identified and their structures determined. The results show that refined structures obtained using automatically collected SerialRED data are indistinguishable from those collected manually using the cRED technique. At the same time, SerialRED has lower requirements of expertise in transmission electron microscopy and is less labor intensive, making it a promising high-throughput crystal screening and structure analysis tool.

## Abstract 2

Serial rotation electron diffraction enables fully automated screening and data acquisition on submicrometre-sized crystals using the continuous rotation method via combined image processing and control of the transmission electron microscopy hardware. A data processing pipeline revolving around hierarchical cluster analysis deals with the large multi-crystal data set for phase analysis and structure determination.

## 1. Introduction   

Understanding the arrangement of atoms in solids, whether of materials science or life science interest, is often key to understanding their nature and function. However, it is sometimes difficult to grow crystals large enough (>5 × 5 × 5 µm) for structure determination using conventional single-crystal X-ray diffraction (SCXRD) techniques. Recent developments of new electron crystallographic methods, especially three-dimensional single-crystal electron diffraction (SCED), provide unique opportunities for structure determination of nano- and micrometre-sized crystals that are too small to be studied by conventional SCXRD. Over the last decade, the capability of electron diffraction (ED) for structure determination has been demonstrated on a large variety of samples, for example inorganic (Kolb *et al.*, 2010 ▸; Jiang *et al.*, 2011 ▸; Martínez-Franco *et al.*, 2013 ▸; Smeets *et al.*, 2013 ▸, 2014 ▸; Su *et al.*, 2014 ▸), organic (Kolb *et al.*, 2010 ▸; Gorelik *et al.*, 2012 ▸; Zhang *et al.*, 2013 ▸; Gruene *et al.*, 2018*b* ▸; Jones *et al.*, 2018 ▸) and protein crystals (Shi *et al.*, 2013 ▸; Yonekura *et al.*, 2015 ▸; Clabbers *et al.*, 2017 ▸; Xu *et al.*, 2018 ▸; Lanza *et al.*, 2019 ▸). For this reason, the method has recently attracted a great deal of attention (Brown & Clardy, 2018 ▸).

The three-dimensional SCED methods in transmission electron microscopy (TEM) that have emerged mimic the well established SCXRD methods. A set of ED patterns is collected by rotating the crystal inside a TEM using the goniometer system while sampling the reciprocal space. The sampling methods have evolved from stepwise goniometer rotation alone (Kolb *et al.*, 2007 ▸; Shi *et al.*, 2013 ▸), combined with a precession of the electron beam at every step (Kolb *et al.*, 2008 ▸) or with fine step-wise electron beam tilt (Zhang *et al.*, 2010 ▸; Wan *et al.*, 2013 ▸), to nowadays widely used continuous goniometer rotation (Nederlof *et al.*, 2013 ▸; Hattne *et al.*, 2015 ▸; Gemmi *et al.*, 2015 ▸; Wang *et al.*, 2018*b* ▸,*a* ▸). The latter is based on the continuous rotation method in X-ray crystallography (Arndt & Wonacott, 1977 ▸). The benefit of continuous rotation electron diffraction (cRED) is that data collection times are greatly reduced (to less than a few minutes) and that the reciprocal space is fully sampled, with the exception of some small wedges of missing data during the read-out of the detector (depending on the model). The data can be processed using programs developed either specifically for ED, such as *ADT* (Kolb *et al.*, 2007 ▸), *PETS* (Palatinus, 2011 ▸) and *RED* (Wan *et al.*, 2013 ▸), or for X-ray diffraction, such as *XDS* (Kabsch, 2010 ▸) and *DIALS* (Winter *et al.*, 2018 ▸). After reconstruction of the three-dimensional reciprocal lattice, the unit cell can be determined and diffraction intensities can be extracted for structure determination using the existing programs developed for X-ray diffraction such as *SHELX* (Sheldrick, 2008 ▸). All of the above methods have shown great ability in terms of structure determination of nano-sized crystals (Yun *et al.*, 2015 ▸).

Because of the development of fast data collection by cRED, there is a strong need for automation of data collection and data processing. At present, the cRED data collection is still very much a manual and time-consuming endeavor; most of the time is spent finding suitable crystals. In addition, the selection of crystals can be subject to human bias. The associated risk is that crystals with different orientations needed to obtain complete three-dimensional data or eventual new minor phases in multi-phase samples are ignored. Compared with powder X-ray diffraction (PXRD), the subjectivity in sample selection and representability of the bulk material are well known drawbacks of using TEM. Although phase analysis and structure determination have been successfully demonstrated in multiphasic samples, it has been very tedious and time-consuming (Yun *et al.*, 2014 ▸). Automated crystal screening and selection would help to alleviate such drawbacks and make increasing the through-put of data collection possible (Cichocka *et al.*, 2018 ▸). Furthermore, we have previously shown that merging datasets from multiple crystals not only increases the data completeness, but also improves the data quality and therefore the resulting structural model (Xu *et al.*, 2018 ▸).

Meanwhile, modern TEMs are uniquely positioned to enable fully automated experiments. Recently, inspired by the serial crystallography method employed at large scale facilities (Chapman *et al.*, 2011 ▸; Stellato *et al.*, 2014 ▸), we developed serial electron diffraction (SerialED) (Smeets *et al.*, 2018*b* ▸) and the software *Instamatic* (Smeets *et al.*, 2018*a* ▸) using Python (Python Software Foundation, https://www.python.org/) as an alternative method of collecting ED data. The SerialED method combines computer-controlled stage translation with beam shift to automatically collect diffraction data on a large number of crystals. We previously explored the use of SerialED data in the context of structure determination (Smeets *et al.*, 2018*b* ▸) and quantitative phase analysis (Smeets *et al.*, 2019 ▸). However, indexing a randomly oriented diffraction pattern in SerialED data has been challenging, because it is difficult to accurately determine the crystal orientation. On the other hand, it is straightforward to determine the unit cell and index reflections using cRED data. It followed naturally to combine SerialED with cRED to realize automated data collection and direct data reduction using the well established software for X-ray crystallography.

We have recently implemented several steps towards automated cRED collection in *Instamatic*, including automatic screening for crystals by image recognition (Smeets *et al.*, 2018*b* ▸), identification of suitable crystals via diffraction pattern analysis by machine learning and manual crystal tracking by defocusing every *n*th diffraction pattern during crystal rotation (Cichocka *et al.*, 2018 ▸). In this work, we present our efforts to fully realize multi-crystal cRED data acquisition without human intervention in line with further development of the software *Instamatic* (Smeets *et al.*, 2018*a* ▸). In this method, which we refer to as SerialRED, crystals are automatically identified and spotted, looping over a pre-defined raster grid. For each suitable crystal, cRED data are collected (Fig. S1 of the supporting information) using an automatic tracking routine. In tandem, we developed a data reduction pipeline to deal with the large number of datasets collected, focusing on the ensemble rather than individual datasets. Hierarchical cluster analyses (HCAs) (Giordano *et al.*, 2012 ▸; Foadi *et al.*, 2013 ▸) serve to make the optimal selection for data merging as not all crystals may diffract equally well, but also to deal with multi-phase materials. We demonstrate the application of SerialRED on three zeolite samples (a pure zeolite, a mixture of two zeolites and a zeolite containing an impurity) and one metal–organic framework sample. We show that using data collected with SerialRED and processed using our in-house developed data processing pipeline, the different phases present in the sample can be grouped and identified automatically. The structures of all tested samples could be successfully solved *ab initio* from the data merged from a number of optimal SerialRED datasets as judged by the HCA.

## 2. Experimental setup for automated SerialRED data collection   

In principle, the method is applicable to all modern TEMs, provided that the programming interfaces for TEM control and camera control are accessible. In our case, the method was implemented on a JEOL 2100 LaB_6_ TEM with a fast direct-electron detector, ASI Timepix (55 × 55 µm pixel size, model QTPX-262k). A small condenser lens aperture (the second smallest on the JEOL TEM) and a large spot size (5) are used globally for reduced beam illumination and better coherence. A continuous carbon-film supported TEM grid (CF400-Cu-UL grids from Electron Microscopy Sciences) is used in order to minimize the confusion for the crystal finding algorithm.

A key factor that hinders automation of cRED data collection is the crystal movement during rotation because of the mechanical design of the goniometer. Previous studies in electron imaging tomography show that the eucentric height changes from point to point, but can be predicted using a simple geometric rotation model (Zheng *et al.*, 2004 ▸). In diffraction mode, the position of the crystal is obscured and not accessible without changing the lens settings. The problem is exacerbated by the continuous rotation during data collection, so that any tracking algorithms must be dynamic and quick to apply. We recently started experimenting with a function to automatically apply a large defocus to the diffraction pattern using the intermediate lens (IL1 on a JEOL microscope) at regular intervals (*i.e.* every *n*th frame) which generates an image of the crystal in the primary beam. The crystal is kept in the center of the primary beam by manually adjusting the stage translation during rotation (Cichocka *et al.*, 2018 ▸). In SerialRED, crystal tracking is performed by analyzing the image in the defocused primary beam, calculating the crystal displacement, and then translating the electron probe using the beam shift deflectors (CLA1). To stabilize the position of the primary beam in the diffraction patterns, we apply a descan using the two sets of image shift deflectors (IS1 and IS2). Therefore, we have calibrated the ratios between the corresponding deflector values (CLA1, IS1 and IS2) in both focused and defocused conditions. We also calibrated the sample stage position, because stage translation is used to center the crystals on the screen before data collection starts to minimize the beam shift required.

**Figure 1:**
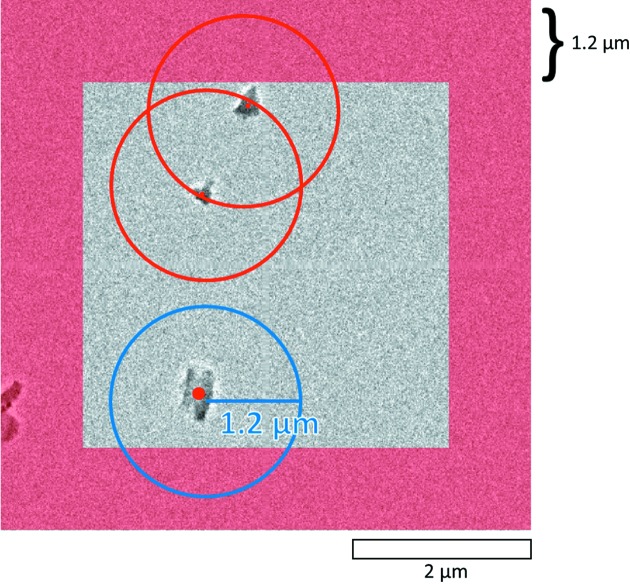
Selection of suitable isolated crystals for data collection. If two crystals are closer than a pre-defined threshold distance (in this case 1.2 µm), they will be both labeled *not isolated*. Among the three crystals found in this figure, two of them (at the centers of the red circles) are too close to each other (within the 1.2 µm area). Therefore, only the crystal inside the blue circle passes the criteria for data collection. Any crystal inside the red masked region is considered to be too close to the edge of view, and is first moved to the center using stage translation before applying the selection criteria again. Both thresholds can be tuned.

To begin the experiment, the user defines a region to scan for crystals. This region (often in the order of 100 s of µm in *x* and *y*) is converted to a set of raster coordinates to loop the stage position over (Fig. S1). Typically, at every 10th position, an automatic adjustment of the eucentric *Z* height is carried out in order to minimize the crystal movement during rotation. At each stage position, an overview image is taken using a parallel probe at a low magnification (*i.e.* 2500×) to locate suitable crystals. This is done using an adaptive threshold algorithm (Smeets *et al.*, 2018*b* ▸); suitable crystals are picked out if they meet the criterion of being ‘isolated’ (Fig. 1 ▸). To avoid large changes of the deflector values which may cause distortion of diffraction spots and problems for crystal tracking, the crystals are first roughly centered by stage translation. The electron probe is then slightly converged to 1 µm in diameter and translated to the crystal with beam shift, and cRED data collection is conducted automatically.

**Figure 2:**
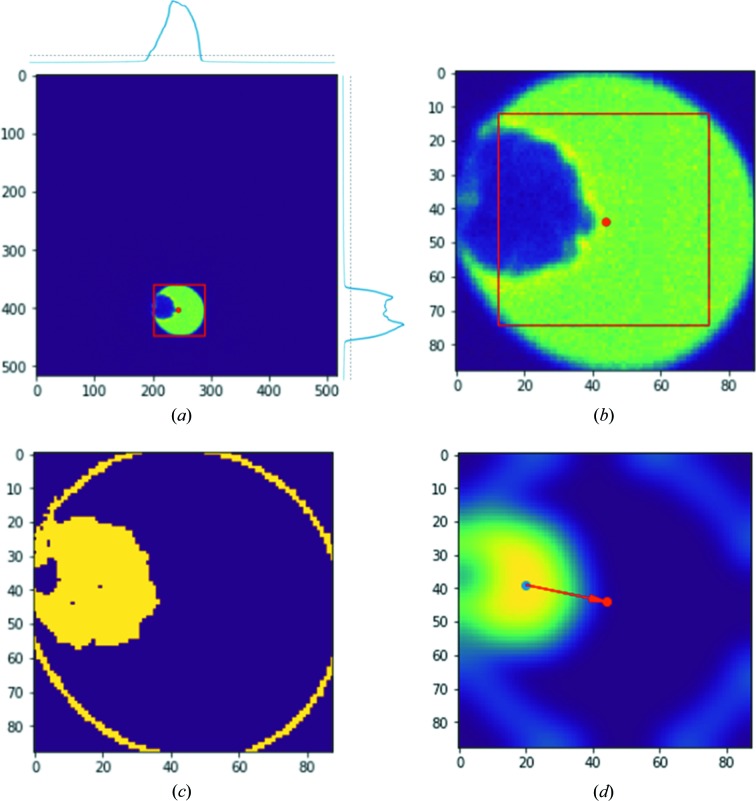
(*a*) Calculation of the beam center from a defocused diffraction image. The diffraction image is summed in the *x* and *y* directions as shown at the top of and on the righthand side of the image, where the dotted line shows the threshold above which the corresponding pixels are considered ‘bright’. These define a bounding box around the defocused primary beam. (*b*) Cropped defocused diffraction pattern for image analysis. The position of the primary beam (defined as the center of the cropped image) is indicated by a red dot. The red squared area is used for image variance calculation using Equation (S4). (*c*) Segmented binary image of (*b*) with intensity cut-off percentiles of 10 and 20%, implemented by taking the third and fourth bins of a 20-binned intensity histogram from the image in (*b*). The yellow ring in (*c*) corresponds to the gradient between the primary beam and the background. (*d*) Blurred image of (*c*) by applying a two-dimensional Gaussian filter. The blue dot in (*d*) shows the calculated crystal center and is used for calculating the beam shift to apply (the vector between the blue and red dots).

The crystal tracking procedure during data collection is performed on a regular basis by defocusing the diffraction pattern and tracing the crystal movement *via* a particle recognition algorithm. For each defocused image, the center of the defocused primary beam is calculated and the bright area is cropped out for faster image analysis [Fig. 2 ▸(*a*)]. The intensity variation of the cropped area [marked by a red square in Fig. 2 ▸(*b*)] is used to determine the position of the particle in relation to the center of the beam. The position of the particle is computed by selecting the pixels with intensities corresponding to the contrast of the crystal, *i.e.* the dark pixels outside the defocused probe and the bright pixels corresponding to the carbon film are ignored. Fig. 2 ▸(*c*) shows that this always results in an intensity gradient at the edge of the defocused primary beam. Subsequently, a Gaussian filter is applied and the pixel with the highest intensity in the filtered image [Fig. 2 ▸(*d*)] then corresponds to the center of the particle. In this way, the influence from the beam edge is eliminated. By taking the difference vector between the beam center and particle center, the appropriate change of the beam shift deflector can be calculated and applied to re-center the particle in the electron probe (Fig. S2). It is important to note that during this analysis, the goniometer is still under rotation so that the correction of crystal movement should be fast enough to compute and apply. To correct for the movement of the crystal, beam shift is preferred over stage translation because stage translation is relatively slow and has some errors caused by the backlash (∼200 nm) in the stage position, which are comparable to the required stage translation, normally <500 nm.

Because beam shift will cause a shift of the primary beam that needs to be compensated for, we apply a beam descan below the sample to move the primary electron beam back to the optical axis. This is important to ensure that not only the position of the primary beam is stable (which is required by data processing software *XDS* or *DIALS*), but also the defocus is predictable. On a poorly calibrated system, the primary beam, and consequently, the defocused pattern, can easily move out of the view of the camera. The goal here is to keep both the focused and defocused diffraction images at their original positions as if no beam shift has been applied. This is achieved by applying changes in the image shift deflectors *x* and *y*, which are calculated by solving a set of equations (see equations S5 and S6 of the supporting information). The whole process of automatic crystal tracking and beam descan takes around 0.2 s (mostly depending on the response time of the computer-to-deflector interface). As long as the exposure time per frame is longer than 0.2 s, only one diffraction pattern will be foregone to apply automatic crystal tracking. Therefore, 10% of the cRED data is lost if the crystal tracking is performed every 10th diffraction pattern, with minimal loss in data quality as discussed previously (Cichocka *et al.*, 2018 ▸). After the data collection on one crystal has finished, the next crystal is centered and automated cRED data collection starts again. When the data have been collected on all suitable crystals in the overview image, the stage is translated to the next position for crystal finding. This procedure is repeated until all stage positions have been exhausted.

A typical SerialRED data collection on a JEOL JEM2100 microscope can screen *ca* 500 particles per hour, among which cRED data are collected from suitable crystals. The full details of the experimental implementation can be found in the supporting information and examples are given in Section 4.

## 3. Automated data processing pipeline and hierarchical cluster analysis   

To address the need to process the large number of cRED datasets collected using SerialRED, we have developed an automated data processing pipeline. As part of the data collection routine, for every cRED dataset, the images are written in a compatible format along with the required metadata and instruction files for data processing software, such as *XDS* (Kabsch, 2010 ▸), *DIALS* (Winter *et al.*, 2018 ▸) and *REDp* (Wan *et al.*, 2013 ▸). The data processing pipeline consists of a set of Python scripts developed in our lab, including functions for automatically running *XDS* or *DIALS* on all datasets, extracting lattice parameters and integration statistics, cluster analyses, as well as generating input files for various data processing software. Generally, *XDS* is run in all subdirectories containing the file XDS.INP. Lattice parameters and integration statistics are then extracted from the file CORRECT.Lp (if indexing was successful). We then use HCA (Ward, 1963 ▸) to find the most common unit cell with a lattice clustering method as described by Giordano *et al.* (2012 ▸) and select the optimal datasets for merging using the reflection-based clustering method described by Foadi *et al.* (2013 ▸). The clustering algorithms also serve to group crystals belonging to the same phase and remove possible outliers and wrongly indexed datasets. The scripts for data processing are available at https://github.com/stefsmeets/edtools and are generally applicable in multi-crystal electron diffraction. Examples of the application of automated data processing and HCA are given in Section 4.

### 3.1. Lattice-based clustering   

Unit cell and space group information can be extracted by parsing CORRECT.Lp. We initially implemented the linear cell variation distance proposed by Foadi *et al.* (2013 ▸), but found that simply taking the distance between the lattice parameters
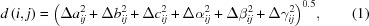
or the volume *V*

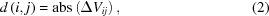
was more effective for our data. Here, *d* corresponds to the distance between unit cells *i* and *j*. A linkage map is calculated from the distance matrix using the ‘average’ method (Sokal & Michener, 1958 ▸). The clusters are visualized by dendrograms, which can be used to define a suitable cut distance for clustering similar unit cells.

For each cluster, six histograms for each of the unit-cell dimensions (*a*, *b*, *c*, α, β, γ) are plotted to visualize the distribution of lattice parameters, inspired by the *cell_explorer* program available in the *CrystFEL* software (White *et al.*, 2012 ▸). By fitting a normal distribution through the histograms, the average lattice parameters and standard deviations can be obtained. An evaluation of the Laue symmetry is performed by grouping the unit cells by their lattice type, and for each group, summing the number of indexed reflections. The idea is that the lattice type with the highest score (most indexed reflections) is the most likely one.

### 3.2. Reflection-based clustering   

The average lattice parameters and Laue symmetry are then used to re-extract the data using *XDS*. If one aims to reduce data for one specific phase, the corresponding unit-cell parameters can be used, and datasets corresponding to other phases will fail. HCA can then be performed on all the datasets that were successfully indexed. The distance metric described by Giordano *et al.* (2012 ▸) is derived from the correlation coefficients of the common reflection intensities (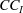) between two datasets *i* and *j*, and defined as
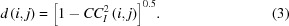



The *CC*_*I*_ values are reported by *XSCALE* in the XSCALE.Lp file. Tuning the resolution range and the *I*/σ in the input can have a large effect on the outcome of the cluster analysis. For cRED data, it is worthwhile to consider limiting the resolution range to where the reflections have been well determined (as indicated by *CC*_1/2_ and *I*/σ) using the INCLUDE_RESOLUTION_RANGE instruction. The same can be achieved by defining the minimum *I*/σ, which we typically set at 2 (*i.e.* MINIMUM_I/SIGMA = 2). We have found the ‘average’ method to be effective for the linking algorithm. The clusters are visualized using a dendrogram to help with the selection of the cut distance. In our data processing pipeline, each of the clusters is output in a separate folder. The data are automatically merged using *XSCALE. POINTLESS* (Evans, 2011 ▸) is run to assess the symmetry of the merged dataset, and the merged reflection data are converted to a format compatible with the *SHELX* suite (Sheldrick, 2008 ▸). Structure solution and refinement are performed using the merged data with existing programs developed for X-ray diffraction.

## 4. Applications   

**Figure 3:**
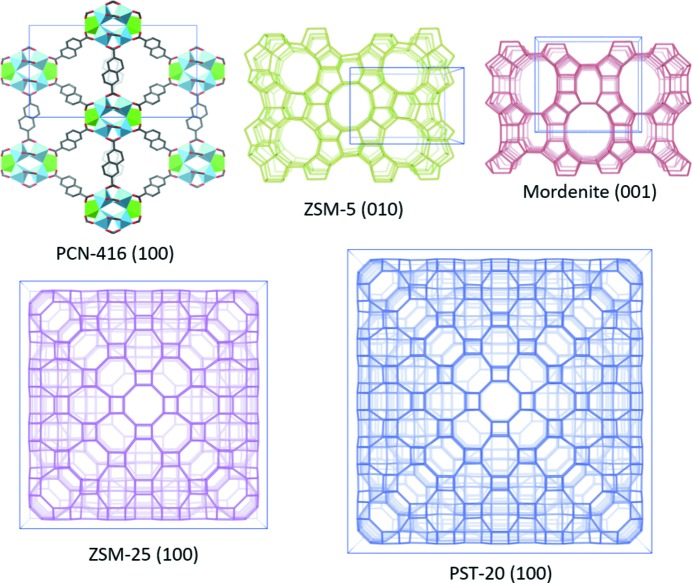
Schematic representations of the framework structures of PCN-416, ZSM-5, mordenite, ZSM-25 and PST-20 that are used in this study. Only *T*–*T* (*T* = Si, Al) connections are shown in the zeolite structures; O atoms have been omitted for clarity.

For testing purposes, data were collected on single-phase samples of aluminosilicate zeolite ZSM-5 (*Pnma*, *a* = 20.07, *b* = 19.92, *c* = 13.42 Å) with framework type **MFI** (Baerlocher *et al.*, 2007 ▸), and the metal–organic framework PCN-416 (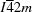, *a* = 16.496, *c* = 29.947 Å) (Yuan *et al.*, 2018 ▸). We were interested to find out if the method could be useful for mixed-phase materials and examined a sample containing two aluminosilicate phases of ZSM-5 and mordenite (**MOR**) (*Cmcm*, *a* = 18.256, *b* = 20.534, *c* = 7.542 Å). We also present a study on a real-world example, the aluminosilicate PST-20 (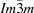, *a* = 55.0664 Å) containing an impurity of ZSM-25 (**MWF**, 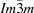, *a* = 45.0711 Å) (Guo *et al.*, 2015 ▸), which are two of the largest zeolite structures found to date. Table 1 ▸ shows the reported structural information and the framework structures of the tested samples are given in Fig. 3 ▸. A summary of the experimental details is given in Table 2 ▸.

### 4.1. ZSM-5   

For development and testing purposes, we worked with a powder of pure calcined ZSM-5 (Kokotailo *et al.*, 1978 ▸). The data presented here on ZSM-5 were collected over a period of 6 h spanning three sessions. In total, 126 datasets comprising at least 5° rotation were collected, out of which 60 could be indexed using *XDS* (Kabsch, 2010 ▸). The unindexed datasets mostly contain blank images (from particles that do not diffract) or very poor diffraction patterns (from particles found in agglomerates).

**Figure 4:**
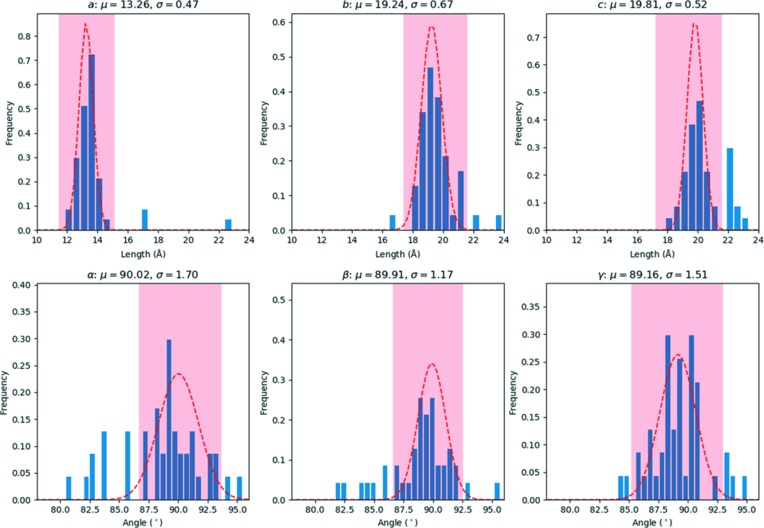
Histograms showing the distribution of the unit-cell parameters obtained from 60 datasets for sample ZSM-5 that were processed using *XDS*. A normal distribution was fitted to the data in the area shaded in red, giving an average lattice of *a* = 13.26 (47), *b* = 19.24 (67), *c* = 19.81 (52) Å, α = 90.0(1.7), β = 89.9(1.2), γ = 89.2(1.5)°.

**Figure 5:**
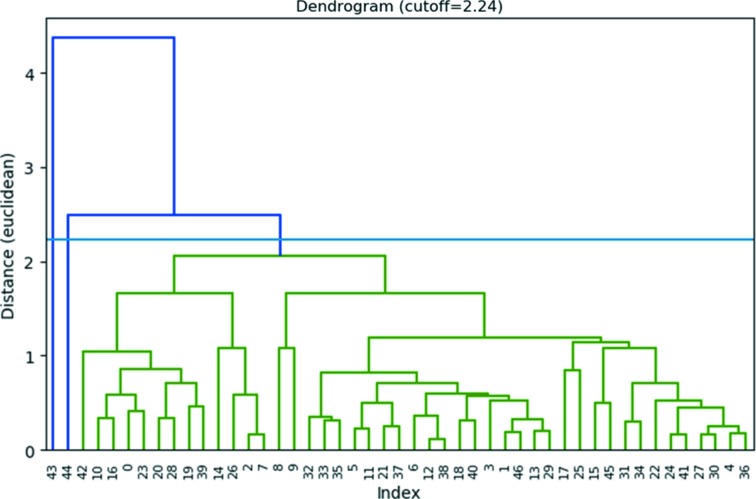
Dendrogram showing the results of the hierarchical cluster analysis of the lattice parameters of ZSM-5, using the Euclidean distance between the lattice parameters as a metric. The cut distance is represented by the blue line (2.24). Two outliers (No. 43 and No. 44) with deviating lattice parameters were excluded, resulting in a single cluster with 45 items.

At first, the average unit cell was found by making a histogram of the cell parameters (Fig. 4 ▸). The average lattice parameters corresponded to *a* = 13.26 (47), *b* = 19.24 (67), *c* = 19.81 (52) Å, α = 90.0(1.7), β = 89.9(1.2), γ = 89.2(1.5)°, which are slightly smaller than the expected orthorhombic cell. A total of 47 datasets could be re-indexed using *XDS* with this unit cell (Table S1), assuming a corresponding Laue symmetry of *mmm* (using space group No. 16, *i.e. Pmmm*). The lattices were analyzed with HCA, using the ‘average’ linkage method and the Euclidean distance between the lattice parameters as a metric (Fig. 5 ▸). Two outliers (No. 44 and No. 45) with deviating lattice parameters were excluded, resulting in a single cluster with 45 items.

**Figure 6:**
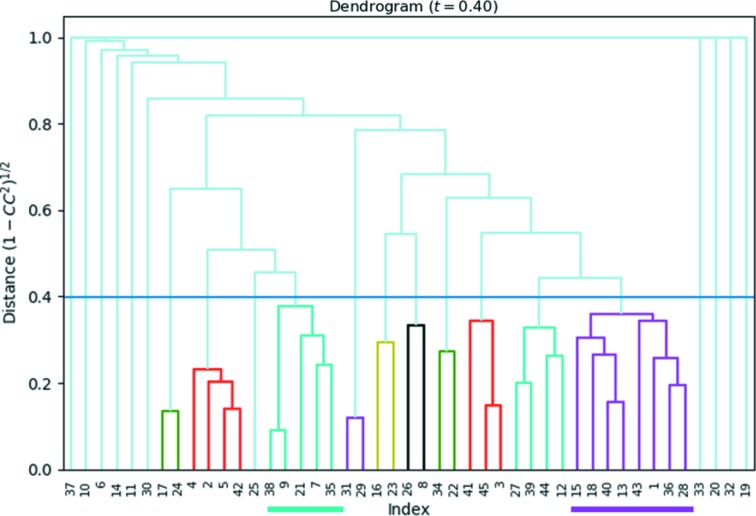
Dendrogram showing the results of the hierarchical cluster analysis of extracted intensities, using the correlation coefficients of the common reflection intensities (*CC_I_*) between pairs of datasets. The cut distance is represented by the blue line at 0.40 (corresponding to *CC_I_* = 0.92). In total, ten clusters were identified. The two largest clusters, in purple (eight datasets) and teal (five datasets), comprised the highest data quality (assessed through completeness and *CC*_1/2_) and were used for structure determination.

Next, we applied reflection-based clustering on this cluster to select and merge the most suitable datasets for structure determination. This was done by running *XSCALE* using all datasets with a resolution limit of 1.2 Å to select only the low-angle reflections for calculating the 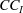 values. Fig. 6 ▸ shows the dendrogram for the HCA using the ‘average’ linkage method. The cut distance was set at 0.4, which corresponds to a 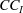 of 0.92. This resulted in ten clusters (Table S2 of the supporting information), out of which clusters 3 and 10 show the highest data completeness while still having reasonable statistics of *R*_meas_ and *I*/σ. For each of the clusters, the corresponding datasets were merged using *XSCALE*, and structure determination was successful using *SHELXT* (Sheldrick, 2015 ▸). We used the ‘–a0.4’ command line instruction in *SHELXT* to allow all centro-symmetric space groups to be evaluated, because the values of α_0_ were typically around 0.31–0.33. *SHELXT* uses α_0_ as a probability indicator to judge if a crystal is centrosymmetric (and thus, if it should search for a center of symmetry), but in our experience, the precision of the ED data is often not sufficient to meet the default threshold of 0.3. For both clusters, the space group was identified correctly by the software, and all framework atoms were revealed and labeled correctly. Interestingly, the re-indexing operators (*a*′ = *b*, *b*′ = *c*, *c*′ = *a* for cluster 3 and *a*′ = *c*, *b*′ = −*b*, *c*′ = *a* for cluster 10) reveal the relation between the two datasets. There is an indexing ambiguity in ZSM-5 because the lattice parameters for *b* and *c* are very close. The indexing ambiguity is a well known problem when merging data from a large number of randomly oriented crystals (Brehm & Diederichs, 2014 ▸). The ZSM-5 framework model was refined against both datasets using *SHELXL* (Sheldrick, 2008 ▸). Similarity restraints were applied to the Si—O bonds. The lattice parameters were scaled to match the average Si—O bond length with the expected value of 1.61 Å. All atoms were refined anisotropically using rigid-bond restraints (RIGU) to maintain reasonable ADPs. The refinements were stable and converged with *R*1 = 0.218, *wR*2 = 0.5448 and *S* = 1.41 for cluster 3, and *R*1 = 0.238, *wR*2 = 0.5875 and *S* = 1.60 for cluster 10. Refinement details can be found in Tables 3 ▸ and S3.

### 4.2. PCN-416   

We also tested the method on the metal–organic framework PCN-416. Its framework structure consists of [Ti_8_Zr_2_O_12_(COO)_16_] clusters that are connected *via* 2,6-naphthalenedi­carboxyl­ate (NDC) as the organic linker. The structure of PCN-416 was determined recently with data collected manually using the cRED technique on the same microscope in our lab, which made it a useful sample for comparing the results (Yuan *et al.*, 2018 ▸). SerialRED was run on the sample for 2 h, resulting in 139 datasets, among which 66 were with rotation ranges larger than 5°. Applying the same data processing pipeline as for ZSM-5, the initial average unit-cell parameters [*a* = 16.18 (68), *b* = 16.75 (83), *c* = 18.13 (95) Å, α = 110.94(2.29), β = 113.21(1.72), γ = 94.59(4.48)°] are close to the published result (related by lattice symmetry). With the average unit cell and Laue group *I*422, *XDS* was able to reindex 27 datasets. The extracted diffraction intensities were then used as input for the reflection-based clustering (see Fig. S3). With a cut distance of 0.36, four clusters emerged. The largest cluster (with 12 datasets merged) had the best data completeness of 96.8% and an average *I*/σ value of 3.10, at a maximum resolution of 0.9 Å.

The merged dataset was used for direct structure solution using *SHELXT*. The correct structure with the space group 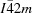 was suggested among five possible solutions with the second lowest initial *R*1 value. The structure solution revealed all atoms in the metal cluster, and 8 out of 12 C atoms of the NDC linker were found directly. This is similar to the published structure determination, where 2 C atoms were missing in the initial solution. In both cases, the positions of the remaining atoms could be deduced easily. Structure refinement using *SHELXL* converged with *R*1 = 0.216, *wR*2 = 0.5351 and *S* = 1.30 (see also Tables 3 ▸ and S4). All atoms were refined anistropically using the RIGU instruction, and similarity restraints were used on the bond distances and angles in the linker. The SIMU instruction was used to restrain the *U*_*ij*_ components of the ADPs of the C atoms. Notably, the data statistics from our SerialRED data are comparable to the published data (Table S7).

### 4.3. ZSM-5 and mordenite mixed sample   

**Figure 7:**
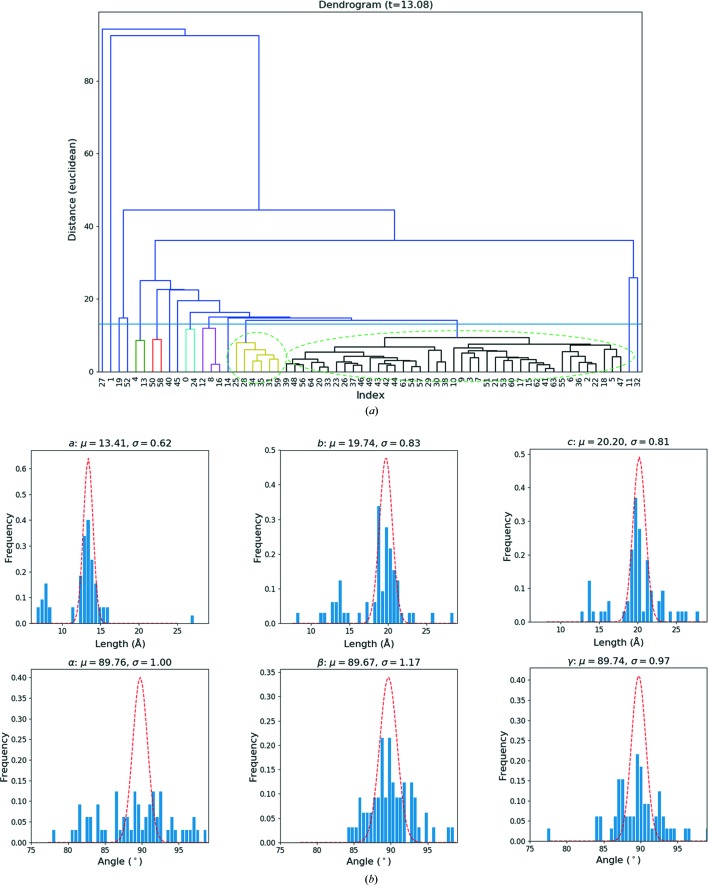

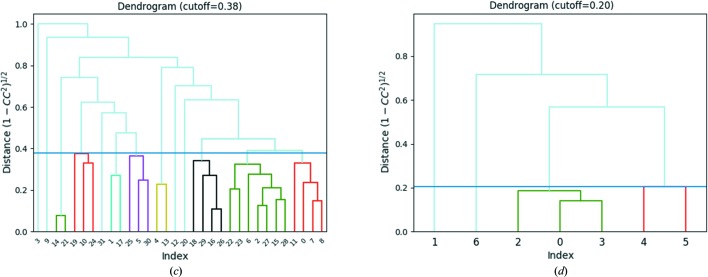
(*a*) Dendogram of the hierarchical cluster analysis of the preliminary unit cells determined by *XDS* using an Euclidean distance for the ZSM-5 and mordenite mixed-phase sample. Two clusters are circled. The unit cells in the larger black clusters correspond to ZSM-5, whereas the smaller yellow cluster corresponds to mordenite. This may also be an indication that there are more ZSM-5 crystals than mordenite in the sample. (*b*) Histogram of the lattice parameters determined by *XDS*. The histograms corresponding to the *a*, *b* and *c* parameters clearly show two peaks that correspond to the two phases. (*c*) Dendrogram of the reflection-based cluster analysis for the datasets with the ZSM-5 lattice. A cut threshold of 0.38 (corresponding to *CC_I_* = 0.92) leads to eight clusters. The largest cluster (green), composed of seven datasets, yielded the combination of the highest data completeness and data consistency (as judged by *CC*_1/2_) and was used for structure determination. (*d*) Dendrogram of the reflection-based cluster analysis for the datasets corresponding to the mordenite lattice. A cut-threshold of 0.2 (*CC_I_* = 0.98) was used to generate two clusters. The red cluster was chosen for structure determination since it has higher data completeness and lower *R*_meas_ (0.13).

To test the application of the method for phase identification and simultaneous structure analysis, we mixed powders of two types of zeolites, ZSM-5 (**MFI**) and mordenite (**MOR**). The SerialRED routine identified 123 suitable crystals over a period of 2 h, out of which 89 comprise a rotation of >5° and 65 could be indexed using *XDS*. We then used these unit cells as the input for the lattice-based HCA. The resulting dendrogram [Fig. 7 ▸(*a*)] clearly reveals two clusters. The larger black cluster (41 cells) corresponded clearly to ZSM-5 [mean unit cell: *a* = 13.51 (77), *b* = 19.82(1.37), *c* = 20.75(1.67) Å, α = 89.60(3.83), β = 89.84(2.52), γ = 89.51(2.49)°], whereas the yellow cluster (6 cells) corresponded to mordenite [mean unit cell: *a* = 7.65 (47), *b* = 13.99(1.23), *c* = 14.36(1.19) Å, α = 82.82(1.98), β = 88.81(2.59), γ = 89.66(1.62)°]. The histograms of the lattice parameters obtained also clearly show two peaks for the unit-cell lengths [Fig. 7 ▸(*b*)].

We then integrated the data using *XDS*, once with the average lattice parameters for ZSM-5 phase (space group *Pmmm*) and once more with those for the mordenite phase (space group *Cmmm*). The idea is, that if the ZSM-5 cell is specified, the indexed datasets should all correspond to ZSM-5 and *vice versa* for mordenite. Here, the space groups correspond to the Laue symmetries of the Bravais lattice types to avoid specifying the space group in *XDS* at this stage, because we also wanted to see if the space groups could be determined from the data themselves. Running *XSCALE* on the 32 ZSM-5 datasets (the other 9 datasets were ruled out at the unit cell clustering step) gave the *CC_I_* values between different datasets, which were used for reflection-based HCA (cut distance = 0.4, corresponding to *CC_I_* = 0.92). The largest cluster, shown in green in Fig. 7 ▸(*c*), had the highest completeness and data quality as judged by *CC_I_*, and was therefore used for structure determination. A similar operation was performed for mordenite, resulting in seven mordenite datasets for the HCA [Fig. 7 ▸(*d*)], where the green cluster consisting of three datasets was picked for structure determination (cut distance = 0.2, corresponding to *CC_I_* = 0.98).

Both structures were solved straightforwardly with merged datasets using *SHELXT*. All Si and O atoms were identified and labeled correctly. In addition, the space groups of the two phases were also determined correctly to be *Pnma* for ZSM-5 and *Cmcm* for mordenite by *SHELXT*. This indicates the high data quality from automated data collection and data merging. The ZSM-5 structure could be refined anisotropically without any geometric restraints against the merged dataset. For mordenite, the refinement was performed isotropically with no restraints because of the lower data completeness that prevented a stable anisotropic refinement. Here, we also scaled the lattice parameters to match the average observed Si—O bond distance with the expected value of 1.61 Å. Crystallographic details of the refinements are shown in Tables 3 ▸ and S5. It was also possible to determine the crystal structures using a few of the single datasets, provided they were of high resolution (ideally <1.0 Å) and completeness.

### 4.4. PST-20 and ZSM-25 mixed sample   

PST-20 is a real-world example of a mixed-phase zeolite, as it can only be synthesized in the presence of significant ZSM-25 impurities at present (Guo *et al.*, 2015 ▸). PST-20 and ZSM-25 share common building units and have some of the largest unit cells found for zeolites to date (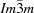, *a* = 55.07 Å for PST-20 and 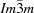, *a* = 45.07 Å for ZSM-25). Both are also rather beam sensitive. To the best of our knowledge, the structures of PST-20 and ZSM-25 were never solved from ED data directly, because of the low data resolution (∼2.4 Å as reported).

Data on the PST-20/ZSM-25 sample were collected over a 4 h session, in which 148 suitable crystals were identified, resulting in 99 datasets with over 5° and 42 over 20° of rotation (Table 2 ▸). A histogram of the rotation ranges for this dataset is shown in Fig. S4a. An initial look at the unit-cell parameters reported by *XDS* indicated that there was indeed a mixture of unit cells, with lattice parameters ranging from 40 to 60 Å, corresponding well to the domains of ZSM-25 and PST-20 (Fig. S4b). In this case, we supplied the correct unit cells and space groups (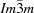) directly to *XDS* for reindexing because of the lower data quality compared with that of the ZSM-5/mordenite sample. This is consistent with our experience with this sample that it is much more beam sensitive than the other zeolites we examined. However, the obtained data resolution (∼1.5 to 2 Å, judging from the *CC*_1/2_ values) is still higher than reported in the previous study. We attribute the improvement in data resolution to the use of a continuous rather than discrete sampling of the reciprocal space.

In total, 29 datasets could be indexed with the unit cell of PST-20, and 15 datasets with that of ZSM-25. The fact that more datasets could be indexed with the parameters of PST-20 is consistent with result from previous work that PST-20 is the main phase of the sample (Guo *et al.*, 2015 ▸). However, 7 datasets could be indexed with both unit cells. This is partly because the structures share common building units and integration with *XDS* is successful as long as at least 25% of the spots are indexed. In this case, we used reflection-based HCA to effectively separate the two phases. The *CC_I_* cut distances for PST-20 and ZSM-25 were 96.9 and 92.5%, respectively. The corresponding dendrograms are shown in Figs. S4c and S4d.

Interestingly, one of the seven common datasets has reasonably high *CC_I_* for both clusters (88.8% for ZSM-25 clusters and over 92.5% correlation for PST-20 clusters, which were initially used as the cut thresholds of the *CC_I_* values for data merging; see the red circled datasets in Figs. S4c and S4d). For that particular dataset, it is more likely to belong to PST-20 because of the higher correlation, so we manually removed that particular dataset from the ZSM-25 cluster. In the end, seven datasets were merged for PST-20, and only two datasets were merged for ZSM-25.

Unfortunately, in our case, the low resolution of 1.5 Å also proved to be a limiting factor for structure determination using *SHELXT*. Therefore, we opted to use the program *FOCUS* (Smeets *et al.*, 2013 ▸; Grosse-Kunstleve *et al.*, 1999 ▸) to see whether it was possible to still determine the structure directly from the merged data. *FOCUS* is a dual-space zeolite-specific program that makes use of chemical information about zeolites (*i.e.* it looks for a three-dimensional four-connected framework with known bond angles and distances) to supplement the diffraction data. In this way, it can make up for the loss of resolution. In total, 29 884 and 30 4521 trials were run for the merged datasets of PST-20 and ZSM-25, respectively. Within around 48 h, 56 structure hits were achieved for PST-20, out of which 55 corresponded to PST-20. For ZSM-25, 236 structures were found in 60 h (of which, 234 match ZSM-25). The determined framework structures matched previous work (Guo *et al.*, 2015 ▸). We performed a preliminary refinement of both structures against the merged datasets. The data were cut at 1.5 Å resolution for both structures according to the *CC*_1/2_ statistics (Karplus & Diederichs, 2012 ▸). Despite the low resolution, with geometric restraints for the Si—O bond lengths (DFIX), O—Si—O bond angles (DANG) and atomic displacement parameters (RIGU), the anisotropic refinements of both structures were convergent and stable. However, considering the relatively low data-to-parameter ratio, in the end the refinements were carried out isotropically (see Tables 3 ▸ and S6) with EADP constraints for the same type of atoms in order to eliminate large ADPs.

## 5. Discussion   

Currently, the rotation ranges achieved by automated data collection are in many cases lower than those with manual tracking. Further optimization of the SerialRED data collection can increase the data ranges. The atomic coordinates obtained through the structure refinements against the SerialRED data are consistent with those established in the literature. It is worth highlighting that the refinement results on ZSM-5 and PCN-416 are virtually indistinguishable from those of the manually collected cRED data based on some of the recent publications on the same materials (Gruene *et al.*, 2018*a* ▸; Wang *et al.*, 2018*b* ▸; Yuan *et al.*, 2018 ▸), in terms of data completeness, number of reflections, *I*/σ values, *R* values, *etc*. That is to say, one cannot really distinguish whether a dataset is collected by a human or by a computer.

However, we did observe that the unit cells obtained from the method may have a certain variation. This is because the sample height is not consistent over the large area of the microscopy grid that we are sampling. The sample height is known to affect the magnification of the diffraction pattern (Ångström *et al.*, 2018 ▸). Averaging a larger number of indexed unit cells can usually give more accurate unit-cell parameters.

The clustering methods play a very important role in the analysis and processing of SerialRED data. It is simply too time-consuming to sort through the data manually, and with so much data it is key to think about the ensemble rather than individual datasets by themselves. Besides, some of the datasets are not useful for further analysis because the data may be collected from a poorly diffracting crystal, an agglomerate or the rotation range may simply be too low. More importantly, the HCA can be used for phase analysis and for identifying minor phases that cannot be detected by powder X-ray diffraction. Furthermore it is useful to find the best matching data for structure refinement.

Phase analysis by HCA is performed in two steps: lattice-based clustering and reflection-based clustering. Firstly, phases/impurities with very different unit-cell parameters can be directly identified using lattice-based clustering. Reflections for each phase are indexed according to the unit cell. Considering the spread in unit-cell parameters often observed for SerialRED data, it is difficult to distinguish phases by lattice-based clustering when their unit-cell parameters are similar. We therefore take a second step using reflection-based clustering to distinguish among those phases based on the correlation (*CC_I_*) of integrated reflection intensities between datasets.

Improved structure refinement can be achieved by combining multiple datasets. The data merging strategy follows the 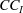 values of the common reflections between datasets. The HCA optimizes the selection of which datasets to merge, thus effectively improving the data quality by ignoring outliers. Clustering methods were initially developed for high-throughput X-ray beamlines at synchrotrons to study biological samples (Giordano *et al.*, 2012 ▸; Foadi *et al.*, 2013 ▸). We find that these methods can be applied equally well in the context of multi-crystal electron diffraction, such as the automated data collections that are the focus of this study, but also when dealing with a large number of manually collected datasets. An important advantage of SerialRED combined with HCA is that minor impurity phases in the specimen, which may not be detectable by X-ray diffraction, can be identified. For unknown phases, their structures can be determined. The number of datasets needed for *ab initio* structure solution depends on the symmetry and initial orientation of the crystal. Based on our experiences, a single dataset from one tiny crystal is enough to solve the crystal structure of a minor phase, provided that the collected data are of sufficient completeness (typically >60%). Combination of multiple datasets can improve the data completeness and reflection intensities, which is important for obtaining accurate atomic positions through structure refinement.

We also find that the method is ready for complex samples. In this study, the PST-20/ZSM-25 sample was solved successfully: both phases were identified and their structures determined. These phases are relatively beam-sensitive, resulting in lowered data resolution, and both have large complex structures that have not been solved directly from the ED data before. By combining automatic data collection and cluster analyses, the resulting data were of good enough quality not only to determine both structures with *FOCUS*, but also for least-squares refinement using *SHELXL*. Despite our efforts in optimizing the integration and data reduction, large *R*1 values were still obtained when refining both structures. We attribute this to the huge differences in reflection intensities and the large fraction of reflections with practically zero intensities because of the presence of common building units in the structures. This phenomenon has previously been identified as one of the limiting factors for direct structure solution for these and related materials (Guo *et al.*, 2015 ▸; Shin *et al.*, 2016 ▸).

At the current stage, the method works best with small and roughly equally sized particles that are relatively sparsely spread over the TEM grid. The minimum size of the crystals that can be used depends on the image magnification. The particle recognition and crystal tracking routine should work as long as the crystals are shown clearly in the image. We normally use a magnification of ×2500, which works well on particles 100–1000 nm in size. Rod-like crystals appear to be harder to work with using the current crystal finding algorithm. This is not limited to the method presented here; in general, such crystals are more difficult to collect cRED data on because the crystal may be in an unfavorable orientation. The current data collection strategy is also not optimized for collecting high-tilt range datasets, since the workflow of data collection sets the starting point of a rotation experiment from wherever the goniometer stopped during the last experiment (see Fig. S5). On our microscope, the rotation speed is set as a microscope constant and cannot be adjusted on-the-fly through the software interface (TEMCOM). Therefore, stage rotation is very time consuming. One could set the starting angle to be very high before starting each rotation experiment (*i.e.* setting the starting angle to always be 50 or −50°). Although this is easily implemented, the drawback of such a strategy is that the data collection efficiency will be sacrificed because a lot of time would be spent on waiting for rotation of the goniometer. It should be noted that on some newer TEM models, the constant rotation speed of the rotation can be controlled very precisely through the software *API*, which is ideal for cRED data collection, and gives additional options to design the experiment. On our JEOL JEM-2100 microscope this function is not available, so we opted for a more pragmatic approach and optimized the rotation speed for data collection of many datasets with smaller rotation. Because the routine mainly operates in the ±40° tilt range at present, the data collection is prone to preferred orientation of the samples, which results in a missing wedge of the data. This is a common problem in ED in particular because continuous carbon film is used and limits the completeness of the data that can be collected. The low completeness for some of the merged data is a consequence of this (Table 3 ▸). A typical histogram of rotation ranges of an automatic SerialRED dataset is shown in Fig. S4a. Although high rotation ranges can be achieved, there are still many datasets with low rotation ranges (<10°). The small rotation ranges may lead to errors in unit cell determination. We attribute these, to a large extent, to the instability of the sample stage, *e.g.* backlash. The backlash problem exists in both the translator and tilt of the stage, which made it difficult to accurately recall the crystal position. Even though many of these datasets can still be used for indexing and structure determination. It is worth mentioning that many of the algorithms can be further improved, *e.g.* for crystal tracking during rotation. There is also a need to improve the mechanical stability of the goniometer system during rotation, which varies among different microscopes and sample holders.

## 6. Conclusions   

In this work, we describe the development of SerialRED, an extension of the serial electron crystallography method (Smeets *et al.*, 2018*b* ▸) by integrating a continuous rotation in combination with an automated tracking routine. The test cases presented show that the data collected can be used to solve complex framework structures of zeolites and metal–organic frameworks *ab initio*, including the determination of the space group symmetry. By applying cluster analysis to the lattice parameters, we were able to automatically distinguish between the different phases in two mixed-phase powders (ZSM-5/mordenite and PST-20/ZSM-25). The structures of each of the phases could then be determined individually by grouping the datasets and selecting the optimal ones for merging using cluster analysis on the common reflection intensities. These include structures of complex zeolites (ZSM-25 and PST-20) that could not be directly solved before using only ED data.

We have outlined the steps required for fully automated data collection, from screening for suitable crystals to automated crystal tracking, and we hope that this work can act as a blueprint to base future experiments on. This offers a major improvement to how ED data are collected, which is often a time-consuming procedure. The fact that structure analysis can be performed using automatically collected multi-crystal ED data opens up new horizons for the characterization of poly and microcrystalline materials. It means that high-quality ED data can be collected with very little human supervision. After initial calibration, the software is fully automated and can run for many hours, or as long as there are crystals on the grid. We show that the method is equally suited for phase identification and structure analysis of complex materials. Of particular note is the application of hierarchical cluster analyses, which are ideally suited to deal with the large number of datasets collected. Using hierarchical cluster analyses, impurities invisible to X-ray diffraction can be detected and identified. Different phases can be grouped using lattice-based clustering. Even phases with similar unit-cell parameters can be distinguished using the reflection-based clustering. The structure of a new phase can be solved from single or multiple cRED datasets, depending on the symmetry of the crystal. The quality of the merged data as assessed through a series of structure refinements is indistinguishable from those collected manually. The SerialRED methods described here are general and can be applied to any nano- and micrometre-sized crystals. With increased interest in organic, pharmaceutical and macromolecular materials, one could think of high-throughput parallel setups to screen for new phases and polymorphs. We expect future studies using SerialRED to show just how versatile the application can be.

## 7. Related literature   

For further literature related to the supporting information, see Koster *et al.* (1992 ▸).

## Supplementary Material

Crystal structure: contains datablock(s) zsm5_cluster3, zsm5_cluster10, zsm5_mixed_phase, mordenite_mixed_phase, pst20_mixed_phase, zsm25_mixed_phase, PCN416. DOI: 10.1107/S2052252519007681/fc5033sup1.cif

Structure factors: contains datablock(s) zsm5_cluster3. DOI: 10.1107/S2052252519007681/fc5033zsm5_cluster3sup2.hkl

Structure factors: contains datablock(s) zsm5_cluster10. DOI: 10.1107/S2052252519007681/fc5033zsm5_cluster10sup3.hkl

Structure factors: contains datablock(s) zsm5_mixed_phase. DOI: 10.1107/S2052252519007681/fc5033zsm5_mixed_phasesup4.hkl

Structure factors: contains datablock(s) mordenite_mixed_phase. DOI: 10.1107/S2052252519007681/fc5033mordenite_mixed_phasesup5.hkl

Structure factors: contains datablock(s) pst20_mixed_phase. DOI: 10.1107/S2052252519007681/fc5033pst20_mixed_phasesup6.hkl

Structure factors: contains datablock(s) zsm25_mixed_phase. DOI: 10.1107/S2052252519007681/fc5033zsm25_mixed_phasesup7.hkl

Structure factors: contains datablock(s) PCN416. DOI: 10.1107/S2052252519007681/fc5033PCN416sup8.hkl

Supporting equations, tables and figures. DOI: 10.1107/S2052252519007681/fc5033sup9.pdf

CCDC references: 1896745, 1896746, 1896747, 1896748, 1896749, 1896750, 1911035

## Figures and Tables

**Table 1:** Tested samples and their reported structure information

Material	ZSM-5†	PCN-416‡	Mordenite§	PST-20¶	ZSM-25¶
Composition	Si_96_O_192_	C_192_O_123.84_Ti_16_Zr_4_	Si_64_O_128_	Si_2640_O_5280_	Si_1440_O_2880_
Space group reported	*Pnma*	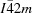	*Cmcm*	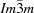	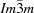
*a* (Å) reported	20.07 (1)	16.496 (3)	18.13 (2)	55.0664 (7)	45.0711 (3)
*b* (Å) reported	19.92 (1)	16.496 (3)	20.49 (2)	55.0664 (7)	45.0711 (3)
*c* (Å) reported	13.42 (1)	29.947 (5)	7.52 (3)	55.0664 (7)	45.0711 (3)
No. of unique atoms	12 Si, 26 O	18 C, 8 O, 2 Ti, 1 Zr	4 Si, 10 O	29 Si, 70 O	16 Si, 40 O

† SCXRD data; Olson *et al.* (1981 ▸).

‡ PXRD data; Yuan *et al.* (2018 ▸).

§ SCXRD data; Meier (1961 ▸).

¶ PXRD data; Guo *et al.* (2015 ▸).

**Table 2:** Experimental details for the tested samples

Sample	ZSM-5	PCN-416	ZSM-5/Mordenite	PST-20/ZSM-25
Data collection time	6 h (3 sessions)	2 h	2 h	4 h
Rotation range (°) (mean, max)	11.86, 76.18	4.04, 44.35†	16.34, 73.60	16.09, 78.46
No. of suitable crystals	250	139	123	148
No. of datasets >5° rotation	126	66	89	99
No. of datasets >20° rotation	43	15	33	42
No. of indexed datasets	47‡	27§	42/11‡	31/19¶

† Collected using a normal single-tilt retainer (±40°); others were collected using a high-tilt retainer (±70°).

‡ Using the average unit cell and corresponding Laue group *mmm*.

§ Using the average unit cell and corresponding Laue group 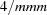.

¶ Using the published unit cell and corresponding space group 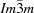.

**Table 3:** Crystallographic data and structure determination details for the tested samples

Phase	ZSM-5 (cluster 3)	ZSM-5 (cluster 10)	PCN-416	ZSM-5 (mixed phase)	Mordenite (mixed phase)	PST-20	ZSM-25
Composition	Si_96_O_192_	Si_96_O_192_	C_192_O_123.84_Ti_16_Zr_4_	Si_96_O_192_	Si_64_O_128_	Si_2640_O_5280_	Si_1440_O_2880_
Space group	*Pnma*	*Pnma*	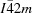	*Pnma*	*Cmcm*	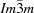	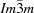
*a* (Å)	20.0 (6)†	20.6 (5)†	16.5 (4)[Table-fn tfn10]	20.4 (6)†	18.1 (3)†	55.07[Table-fn tfn11]	45.07[Table-fn tfn11]
*b* (Å)	20.4 (6)†	19.6 (10)†	16.5 (4)[Table-fn tfn10]	20.1 (8)†	20.0 (0)†	55.07[Table-fn tfn11]	45.07[Table-fn tfn11]
*c* (Å)	13.6 (6)†	13.7 (4)†	29.8 (8)[Table-fn tfn10]	13.6 (4)†	7.7 (4)†	55.07[Table-fn tfn11]	45.07[Table-fn tfn11]
*V* (Å^3^)	5555.5	5542.3	8086.0	5556.0	2790.5	167011.1	91550.9
Structure determination	*SHELXT*	*SHELXT*	*SHELXT*	*SHELXT*	*SHELXT*	*FOCUS*	*FOCUS*
No. of datasets merged	5	8	12	7	2	7	2
No. of data observed (all)	2692 (4734)	3425 (5407)	1254 (2825)	3029 (4488)	555 (904)	1179 (2647)	695 (1492)
No. of parameters	333	333	211	332	44	267	150
No. of restraints	348	348	377	0	0	290	160
Resolution (Å)	0.8	0.8	0.9	0.8	0.8	1.5	1.5
*I*/σ (total)	2.68	2.81	3.10	3.39	3.77	3.76	4.05
*CC*_1/2_ (total)	95.9	91.4	96.8	97.4	98.2	96.1	99.1
Completeness (%)	91.0	96.6	96.8	73.8	56.2	99.7	99.7
*R*1 [*F* ^2^ > 2.0σ(*F* ^2^)]	0.218 (anisotropic)	0.238 (anisotropic)	0.216 (anisotropic)	0.196 (anisotropic)	0.291 (isotropic)	0.490 (isotropic)	0.486 (isotropic)
*R*1(all)	0.260	0.306	0.278	0.227	0.318	0.529	0.527
*wR*2, *S*	0.5448, 1.41	0.5875, 1.60	0.5351, 1.30	0.4735, 1.08	0.6472, 2.16	0.8583, 3.22	0.8448, 3.07

† Averaged from HCA results based on *CC_I_* and scaled with the average Si—O bond length equal to 1.61 Å.

‡ Averaged from the reindexing result (27 datasets).

§ Previously reported by Guo *et al.* (2015 ▸).
