# Supplementary material for: Automated serial rotation electron diffraction combined with cluster analysis: an efficient multi-crystal workflow for structure determination
Source: IUCrJ. 2019 Jul 12;6(Pt 5):854–67. doi: 10.1107/S2052252519007681 (PMC6760450; doi:10.1107/S2052252519007681)
Supplement: Supplementary file 9 [file m-06-00854-sup9.pdf]

IUCrJ

Volume 6 (2019)

Supporting information for article:

Automated serial rotation electron diffraction combined with cluster analysis: an efficient multi-crystal workflow for structure determination

Bin Wang, Xiaodong Zou and Stef Smeets

Raw data that was used in this paper can be found *via* DOI: 10.5281/zenodo.2644205. CIF files containing the refinement details can be found through CCDC numbers: 1896745, 1896746, 1896747, 1896748, 1896749, 1896750 and 1911035.

S1. Implementation details

Modern TEMs allow nearly full control over the goniometer (sample stage), lenses, deflectors, and cameras. This gives substantial control to design and implement new methods, and lends itself well for software automation. We make use of the fact that the TEM can be switched from image mode to diffraction mode *via* software. Automated crystal screening is performed in image mode at low magnification, by translating the sample stage in a raster over a large area, whereas data collection and crystal tracking are performed in diffraction mode, making use of the electron beam deflectors and lenses. In this section, we lay out the steps to implement a fully automated method for multi-crystal electron diffraction with no human supervision. In principle, no bias is introduced in terms of crystal shape for all the crystals on the grid, so the method is considered to be suitable for both pure-phase samples and multi-phase samples.

S1.1. Setup

The experiment is implemented on our JEOL JEM-2100-LaB_6, equipped with a 512 × 512 Timepix hybrid pixel detector (55 × 55 μm pixel size, model QTPX-262k) from ASI. The basic setup for the automatic experiment is to use a small condenser lens aperture. On our TEM, the second smallest condenser lens (physical diameter 50 μm, resulting in an illumination area of 12 μm in diameter when the electron beam is fully spread) is used. In combination with a small spot size (large spot number, 4 or 5 on our microscope) this helps to reduce the intensity of the probe and minimize the convergence angle. In imaging mode, parallel beam illumination is used, but in diffraction mode, the beam brightness (CL3) is tuned to give a probe size of around 1 μm in diameter. The values of the CL3 lens and the corresponding diffraction focus (the intermediate lens, IL1) are recorded. The defocus value should be carefully chosen so that the size of beam in defocused diffraction mode is similar to that in image mode. TEM grid with continuous carbon film (CF400-Cu-UL grids from Electron Microscopy Sciences) is used to avoid any extra confusion for particle recognition algorithm.

S1.2. Deflector and stage calibrations

To track the crystal, the diffraction pattern is defocused at regular intervals. By analyzing the snapshots of the crystal in defocused images, tracking is achieved experimentally by changing the beam shift deflectors (CLA1 in a JEOL TEM), which need to be calibrated in both imaging and

diffraction mode to map out the scale and relative angle between the deflector system and the detector coordinate system. To ensure the diffraction pattern is centered, the image shift deflectors are employed (IS1 and IS2 in a JEOL TEM). Because the diffraction patterns in both focused and defocused conditions need to be taken care of, calibrations for the image shift deflectors are done in both focused and defocused conditions, thus resulting in four additional calibration steps. The calibration methods follow the protocol suggested in a previous paper on SerialED (Smeets, Zou *et al.*, 2018), where 25 points of deflector change (in a 5 by 5 grid) and the corresponding images were recorded. The affine transformation matrix M describing the relation between the deflector values and pixel coordinates is defined as:

$$M = \begin{pmatrix} s_x \cos\theta & -s_y \sin\theta \\ s_x \sin\theta & s_y \cos\theta \end{pmatrix} \qquad \text{(s1)}$$

where the scales s_x and s_y, and the rotation angle θ are obtained from a least-squares fitting between the deflector values (X and Y) and the corresponding pixel shifts in x and y direction as observed using cross correlation with a diffraction pattern taken at the central position. In total, five 2 by 2 matrices, M_{IS1}^{fc}(IS1 in focused condition), M_{IS2}^{fc}(IS2 in focused condition), M_{IS1}^{dfc}(IS1 in defocused condition), M_{IS2}^{dfc}(IS2 in defocused condition), and M_{BS}^{fc}(CLA1 in focused condition), are obtained and used during the tracking procedure. Beam shift calibration (M_{BS}^{Mag}) in real space (*i.e.* MAG1 mode in a JEOL TEM at 2500×) should also be performed.

The stage is calibrated to perform basic centering of interesting particles. The same optimization method is followed by taking a 5 by 5 grid of stage position change under the desired magnification (i.e. 2500×), and using cross correlation to map the pixel coordinates to the corresponding stage translations (M_{stage}).

S1.3. Crystal screening and detection

For crystal screening and detection, we have adapted the algorithms developed previously (Smeets, Zou *et al.*, 2018). In a SerialED experiment, the sample stage is translated in a raster over a large area (typically several hundreds of micrometres), and at each position of the stage, an image is taken at a low magnification (typically 2500×). Crystals are then detected using a local threshold filter, and only isolated crystals are selected. Crystals that are within 1.2 µm from another crystal are discarded, and a centering step is introduced for crystals within 1.2 µm from the edge of the image frame (see Figure 1 & S1). Crystals at the edges are not ideal to work with, because it is unknown what is outside the frame. Therefore, the crystals are first brought to the center of the of the image by stage translation (via M_{stage}). This also serves to minimize the adjustments to the beam position using the deflectors to stabilize the diffraction patterns.

S1.4. Automatic *Z* height adjustment

Setting the correct height of the crystal, as defined by *Z*, minimizes the movement of the crystal during rotation. To automate the height adjustment, an empirical feedback method is used, based on the assumption that the height is already reasonably close to the best value (*i.e.* up to 10 μm away). This assumption is usually valid in a sense that the operator performs the routine TEM alignment before starting the automated experiment. Empirically, at the correct *Z* height, the crystal position (as defined by its pixel coordinates, *x* and *y*) should be invariant of the tilt angle. There are a few ways of adjusting the height automatically. We tried the method described in a previous paper (Koster *et al.*, 1992), but it did not work as perfectly as expected. This is because the method requires calculating the displacement of the image over a certain rotation angle (i.e. 10°). Since we set a global rotation speed (0.86°/s) before the experiment and this cannot be changed during the experiment, the whole *Z* height adjustment process takes very long time since at least 5 points (thus 5 times 10° rotation) need to be sampled. With the existence of backlash in *z* direction, the accuracy of the outcome is also not satisfactory.

Instead, the software sends a command for the goniometer to tilt to a certain degree (a rotation range of 15° is typically used). Every 2 degrees, an image is recorded and compared to the previous image by calculating the difference in positions of the same particle in both images. From how much the position has changed, a decision of *Z* height change of 1 μm is made. *Z* height is adjusted in the way that the movement of the particle is minimized. We are able to achieve similar accuracy for the eucentric height, while the process is much less time-consuming. The height adjustment is made periodically (typically every 10 steps during raster scan) and contributes to improvement of the overall rotation ranges.

S1.5. Crystal rotation and data collection

Stage rotation is also controlled through software. Once a crystal has been identified and centered, the beam is focused on a crystal (with the pre-defined probe size), and the TEM is switched to diffraction mode. When the rotation is initiated, data collection starts with a delay of 0.1 s to avoid any existing backlash and goniometer acceleration in the tilting direction. Backlash may introduce inaccurate readout of the rotation angle, as well as a sudden crystal movement at the very beginning of rotation. Diffraction data are collected typically with an exposure time of 0.5 s. As described in a previous paper (Cichocka *et al.*, 2018), the diffraction patterns are defocused at regular intervals (defined as a defocusing frequency $f_{defocus}$, typically every 10th frame), and an image is taken with a short exposure (typically 0.01 s) to check the crystal position.

Automatic rotation is realized by making use of a tool (Goniotool on a JEOL microscope) that allows us to override the stepping frequency of the motors in the goniometer. In its default state, through the API, the goniometer always rotates at its full speed (~12°/s) to the targeted angle. By reducing the

stepping frequency from 12 kHz to 1 kHz (the lowest allowed value), rotation speed can be reduced to 0.86°/s, which is quite suitable for cRED data collection. Slower rotation also decelerates the crystal movement during rotation, which provides certain tolerance for tracking the crystal.

Because the rotation can only operate at a set speed, we take a pragmatic approach to always initiate stage rotation from the current tilt angle (Figure S5). If the current angle is negative, the target tilt angle is set to 70°, whereas if the rotation is positive, the target angle is set to -70°. Rotation can be interrupted at any time through the JEOLCOM API. This happens when the deflectors hit their limits, or if the crystal moves out of the electron beam.

S1.6. Automatic crystal tracking

The crystal tracking is an essential function for collecting rotation data with high tilt ranges. The defocusing method gives the possibility of chasing the crystal dynamically and tracking the crystal by shifting the electron beam via the beam shift deflectors (Figure S2). The use of the stage position to track the crystal is not preferred for two reasons. First, mechanical backlash makes it difficult to achieve precise positioning of the stage, especially with frequent translations in the sub-micrometer range, and second, mechanical movement is quite slow. We have tested the piezo-stage to control the stage position, and although it is much more precise, it is even slower and the translation is limited to ± 200 nm. In comparison, repositioning the beam via the deflector system is very precise and almost instantaneous.

First, the pixel coordinates of the crystal and the center of the primary beam should be determined. The difference vector between the two positions determines the relative change in the beam shift deflectors to reposition the electron beam. This is done in two steps. First, the bounding box of the primary beam is determined (Figure 2a). The position of the electron beam is then defined as the center of the bounding box. A smaller window (defined as the largest square inside the beam) is used to determine the variance of image contrast (Figure 2b). A histogram of the pixels within the bounding box is used to define the thresholds for segmentation (Figure 2c). A Gaussian filter (using σ = 5) is applied to smooth the binary image. The position of the crystal is then determined as the pixel indices of the most intense pixel (Figure 2d). The difference vector between the beam center and the crystal position is defined as a pixel shift (ΔP) and a beam shift ΔBS is applied to compensate for the difference.

$$\begin{pmatrix} \Delta BS_x \\ \Delta BS_y \end{pmatrix} = (M_{BS}^{Mag})^{-1} \begin{pmatrix} \Delta P_x \\ \Delta P_y \end{pmatrix} \quad \text{(s2)}$$

Here we did assume that the transformation matrix of beam shift in image mode on the detector is the same as that in defocused diffraction mode, which turned out to be valid as long as the defocus value is correctly chosen during the setup step.

On our microscope, the goniometer system is generally more stable at low tilt angles (*i.e.* in the range of ± 40°). Therefore, by starting at higher angles that mark the beginning of a rotation experiment, the movement is exaggerated and more frequent tracking of the crystal position may be needed. Part of this can be attributed to the goniometer design, which makes a conical movement during rotation (*i.e.* the crystal 'swings' around the rotation axis). This prevents eucentric tilting, because the crystal cannot be centered on the rotation axis if it is off the "eucentric height". To get around this, a dynamic tracking algorithm is implemented in the data collection routine. For every focused diffraction pattern, the scale of an image (total intensity over the pixels) is calculated using Equation (s3). Two criteria are employed to verify whether the crystal is still in the electron beam. First, the scale S_{DP} (defined as the total intensity) of every (focused) diffraction pattern is evaluated as:

$$S_{DP} = \sum_{i,j} I_{ij} \qquad \text{(s3)}$$

where i and j are the dimensions of the image. If the scale between two subsequent frames changes by more than a threshold value (*i.e.* $\frac{S_{DP}(n)}{S_{DP}(0)} < 0.85$, where n is the frame number), this is an indication of the crystal moving out of the beam. In an attempt to prevent this, the defocus frequency is increased to 2, to more frequently apply deflector shifts to track the crystal back with the beam (so-called "robust tracking"). A second criterion is defined as the variance of the defocused diffraction pattern:

$$\sigma_{dfc}^2 = Var(defocused\ image) = \sum_{i,j} (I_{ij} - \bar{I})^2 \qquad \text{(s4)}$$

where $\bar{I}$ is the average intensity value over the squared image in Figure 2b. Similarly, robust tracking feature is activated when $\frac{\sigma_{dfc}^2(n)}{\sigma_{dfc}^2(0)} < 0.5$, where n is the frame number. The feature is deactivated after five attempts by resetting $S_{DP}(0)$ and $\sigma^2_{defocus}(0)$ values to the current ones, since in some cases the rotated sample causes permanent change of the scale or variance. When the crystal moves out of the beam completely ($\frac{\sigma_{dfc}^2(n)}{\sigma_{dfc}^2(0)} < 0.2$), or any of the other stopping criteria is hit, rotation is interrupted and data are written automatically to formats compatible for data processing in REDp (Wan *et al.*, 2013), XDS (Kabsch, 2010), and DIALS (Winter *et al.*, 2018).

S1.7. Descan

Whenever a beam shift ΔBS (ΔBS_x, ΔBS_y) is applied, the centers of both the focused and defocused diffraction patterns are also shifted. From the calibration we know that the effect of beam shift on the

diffraction pattern pixel shift would be $M_{BS}^{fc} \cdot \Delta BS$. The effect on the defocused diffraction pattern pixel shift (ΔDD = (ΔDD_x, ΔDD_y)) is calculated on-the-fly every time when applying defocus, which is the difference of the pixel positions of the two beam centers in the defocused images in Figure 2a. A combination of the image shift deflectors ($\Delta IS1$ = ($\Delta IS1_x$, $\Delta IS1_y$) and $\Delta IS2$ = ($\Delta IS2_x$, $\Delta IS2_y$)) are used to ensure the position of the primary beam is stable, which is an assumption made for data processing in XDS and DIALS.

$$M_{IS1}^{fc} \begin{pmatrix} \Delta IS1x \\ \Delta IS1y \end{pmatrix} + M_{IS2}^{fc} \begin{pmatrix} \Delta IS2x \\ \Delta IS2y \end{pmatrix} = -M_{BS}^{fc} \begin{pmatrix} \Delta BSx \\ \Delta BSy \end{pmatrix} \qquad \text{(s5)}$$

$$M_{IS1}^{dfc} \begin{pmatrix} \Delta IS1x \\ \Delta IS1y \end{pmatrix} + M_{IS2}^{dfc} \begin{pmatrix} \Delta IS2x \\ \Delta IS2y \end{pmatrix} = -\begin{pmatrix} \Delta DDx \\ \Delta DDy \end{pmatrix} \qquad \text{(s6)}$$

Eq. (s5) describes that the change of image shift deflector parameters (*ΔIS1, ΔIS2*) should eliminate the shift introduced by the change of beam shift (*ΔBS*) on the focused diffraction pattern. Eq. (s6) is the analog of Eq. (s5) in defocused condition. Eq. (s5) and (s6) then form a system of four linear equations and we solve for the values of *ΔIS1* and *ΔIS2.* Applying this method ensures that the position of the primary beam is stable within a few pixels (up to 5) in general. In a post-processing step, the images can be realigned to higher precision using, for example, cross correlation if this is necessary.

Table S1 Data quality indicators for the cRED data collected on ZSM-5 and indexed in Laue group *mmm*. A default resolution cut-off of 0.8 Å is used globally for all the data sets. This led to various kinds of data statistics which can be seen in the table. A low $I/\sigma(I)$ value or $CC_{1/2}$ value (usually with high R_{meas}) suggests that the actual resolution of the data set is lower than 0.8 Å; a low $CC_{1/2}$ value can also indicate that rotation range for this particular data set is low, leading to too few indexed reflections to get a statistically significant estimate of $CC_{1/2}$ values.

#	d_{max} (Å)	d_{min} (Å)	Total	Unique	Compl. (%)	$I/\sigma(I)$	R_{meas}	$CC_{1/2}$	$I/\sigma(I)^{asym.}$	$B_{overall}$
**1**	4.34	0.80	867	557	7.7	2.90	128.5	5.6	-	4.78
**2**	9.59	0.80	7799	3085	53.8	1.38	24.5	97.1	3.96	7.14
**3**	7.61	0.79	8089	3752	53.5	1.02	47.9	82.1	2.03	4.71
**4**	7.28	0.80	5052	2726	48.8	1.45	40.3	82.5	2.27	5.44
**5**	9.15	0.80	4370	2169	42.2	0.63	106.0	9.1	1.17	4.28
**6**	11.31	0.80	4512	1489	24.9	2.83	21.3	96.8	3.86	5.80
**7**	7.97	0.80	4900	2072	37.3	1.83	24.4	97.2	3.32	6.54
**8**	6.46	0.81	1013	654	9.9	3.53	10.0	99.5	10.06	6.99
**9**	7.29	0.80	8598	3229	55.5	0.37	80.5	76.7	1.53	5.86
**10**	2.81	0.95	167	125	3.2	11.89	66.8	71.6	-	5.82
**11**	5.77	0.80	2951	1500	25.5	2.87	21.3	96.3	3.99	4.83
**12**	6.77	0.80	4132	1670	27.4	2.20	31.2	91.8	2.69	4.78
**13**	6.67	0.81	2017	960	17.1	2.40	27.9	93.5	2.81	4.99
**14**	5.69	0.81	918	693	12.1	3.09	14.5	98.8	5.16	5.03
**15**	4.57	0.80	1323	771	11.0	1.98	25.6	94.8	3.66	5.39
**16**	9.47	0.80	9575	3345	61.6	2.43	20.6	97.2	4.86	6.59
**17**	7.52	0.80	4399	2634	42.5	2.63	23.0	95.5	4.16	4.95
**18**	8.62	0.80	4098	2105	40.2	3.04	16.1	98.3	5.66	5.74
**19**	10.66	0.80	8789	3739	66.6	0.69	99.3	23.4	1.21	3.71
**20**	4.97	0.80	1143	601	9.4	3.12	15.4	98.8	6.01	5.22
**21**	4.09	0.80	1064	812	12.7	3.02	9.3	99.8	11.89	5.85
**22**	13.48	0.80	1996	1032	17.7	3.80	15.3	98.2	5.90	4.29
**23**	7.72	0.80	1032	673	12.8	2.50	13.8	99.6	5.62	5.66
**24**	8.90	0.81	139	131	2.3	8.13	24.4	99.6	-	4.86
**25**	10.73	0.80	2648	1623	30.8	2.34	23.2	96.8	3.41	4.58
**26**	7.35	0.80	2321	1154	22.9	1.65	39.7	90.2	2.06	4.89
**27**	6.02	0.81	535	438	6.5	6.64	50.4	65.4	-	6.36
**28**	5.70	0.80	936	672	13.4	1.47	31.0	93.1	2.69	5.00
**29**	9.18	0.80	983	729	11.4	3.08	17.9	95.9	5.53	6.59
**30**	10.96	0.80	972	641	11.6	1.97	22.6	97.7	5.02	5.57
**31**	2.98	0.81	112	104	2.1	11.57	8.4	99.8	-	3.38
**32**	9.53	0.80	1631	702	14.4	2.89	20.6	98.2	3.73	4.84
**33**	7.30	0.80	2352	1308	22.6	0.44	72.7	67.6	1.86	5.95
**34**	9.43	0.80	7917	3441	61.1	2.24	24.7	96.8	4.41	5.65
**35**	5.12	0.81	295	239	4.8	6.69	31.5	92.9	-	5.07
**36**	9.73	0.80	6954	2736	48.3	1.63	43.5	82.7	2.44	5.26
**37**	10.47	0.80	973	462	9.2	4.34	15.1	96.7	8.52	5.47
**38**	9.62	0.80	7653	3461	59.4	2.81	23.6	96.7	4.58	5.17
**39**	13.18	0.80	6609	2959	52.7	2.48	23.7	95.9	5.15	5.66
**40**	7.55	0.81	1211	689	11.0	2.25	20.9	95.6	5.95	5.09
**41**	1.98	0.82	124	99	2.0	6.99	36.2	80.1	-	2.73
**42**	7.68	0.80	2687	1372	27.0	2.76	17.7	98.5	5.11	5.57
**43**	3.21	0.81	78	71	1.1	4.36	144.3	-58.9	-	5.02
**44**	3.91	0.81	769	455	5.9	1.15	145.9	-1.9	-	4.68
**45**	13.45	0.80	5720	2615	44.1	1.96	26.4	93.5	3.30	5.41
**46**	8.67	0.80	7984	3435	64.2	2.39	22.3	97.4	4.00	5.30
**47**	11.03	0.80	4999	2935	52.7	1.54	32.5	91.4	3.01	5.40

Table S2 Table showing the merging statistics from the reflection-based clustering for ZSM-5.

#	*N*(clustered)	$CC_{1/2}$	*N*(observed)	*N*(unique)	Compl. (%)	R_{meas}	d_{min} (Å)[1]	$I/\sigma(I)$
**1**	2	95.6	654	376	13.8	0.20	1.00	2.45
**2**	4	97.1	6787	2030	66.9	0.25	1.00	2.08
**3**	5	96.3	10360	2729	93.2	0.28	1.00	3.42
**4**	2	81.5	3043	1520	51.4	0.39	1.00	2.04
**5**	2	94.9	2970	1693	51.4	0.22	1.00	2.96
**6**	2	88.4	4651	1831	69.5	0.40	1.00	2.38
**7**	2	80.2	5810	2446	69.5	0.44	1.00	1.71
**8**	3	94.9	6914	2337	63.2	0.30	1.00	2.88
**9**	4	93.2	5337	1125	36.3	0.27	1.00	4.55
**10**	8	93.2	21588	3102	98.4	0.39	1.00	3.45

[1]The resolution was cut at 1.0 to produce this table

Table S3 Crystallographic details for the two clustered data sets of ZSM-5.

	**Crystal data**	
Sample	ZSM-5 (cluster 3)	ZSM-5 (cluster 10)
Formula	[$Si_{96}O_{192}$]	[$Si_{96}O_{192}$]
Formula Weight (g/mol)	5768.64	5768.64
Crystal System	orthorhombic	orthorhombic
Space group	$Pnma$ (No. 62)	$Pnma$ (No. 62)
a, b, c (Å)	20.0(6), 20.4(6), 13.6(6)	20.6(5), 19.6(1.0), 13.7(4)
α, β, γ (°)	90, 90, 90	90, 90, 90
V (Å^3)	5555.5	5542.3
ρ (g/cm^3)	1.72	1.73
F(000)	2880	2880
	**Data collection**	
Temperature (K)	293	293
Radiation (Å)	electrons, 0.02508	electrons, 0.02508
Number of crystals	5	8
d_{min}, d_{max} (Å)	0.77, 10.73	0.79, 11.06
Dataset (h, k, l)	-23→23, -24→24, -16→16	-25→25, -23→23, -16→16
Tot., Uniq. data, R_{int}	17584, 4734, 0.253	37050, 5407, 0.352
Observed Data [$I > 2.0\sigma(I)$]	2692	3425
	**Refinement**	
$N_{reflections}$, $N_{parameters}$, $N_{restraints}$	4734, 333, 348	5407, 333, 348
$R1$ [$F^2 > 2.0\sigma(F^2)$], $wR2$, S	0.2178, 0.5448, 1.41	0.2385, 0.5875, 1.60
Δ/σ (max., avg.)	0.02 (EXTI), < 0.01	0.13 (EXTI), <0.01
$\rho_{min,}$ ρ_{max} (V/Å^3)	-0.20, 0.39	-0.33, 0.23

Table S4 Crystallographic details for the clustered data set of PCN-416.

**Crystal data**	
Sample	PCN-416
Formula	$[C_{192}O_{96.16}Ti_{16}Zr_4]$
Formula Weight (g/mol)	4975.28
Crystal System	tetragonal
Space group	$I\bar{4}2m$ (No. 121)
a, *b*, *c* (Å)	16.5(4), 16.5(4), 29.8(8)
α, *β*, *γ* (°)	90, 90, 90
V (Å^3)	8113
ρ (g/cm^3)	1.020
F(000)	2433
**Data collection**	
Temperature (K)	293
Radiation (Å)	electrons, 0.02508
Number of crystals	12
d_{min}, d_{max} (Å)	0.90, 8.45
Dataset (*h*, *k*, *l*)	-18→18, -18→18, -32→32
Tot., Uniq. data, R_{int}	11248, 2825, 0.229
Observed Data [$I > 2.0\sigma(I)$]	1254
**Refinement**	
$N_{reflections}$, $N_{parameters}$, $N_{restraints}$	2825, 211, 377
$R1$ [$F^2 > 2.0\sigma(F^2)$], $wR2$, S	0.2163, 0.5351, 1.30
Δ/σ (max., avg.)	< 0.01, < 0.01
ρ_{min}, ρ_{max} (V/Å^3)	-0.26, 0.32

Table S5 Crystallographic details for the two clustered data sets of ZSM-5 & mordenite mixed phase sample.

	**Crystal data**	
Sample	ZSM-5 (mixed phase)	Mordenite (mixed phase)
Formula	[$Si_{96}O_{192}$]	[$Si_{64}O_{128}$]
Formula Weight (g/mol)	5768.64	3845.38
Crystal System	Orthorhombic	orthorhombic
Space group	$Pnma$ (No. 62)	$Cmcm$ (No. 63)
a, b, c (Å)	20.4(6), 20.1(8), 13.6(4)	18.1(3), 20.0(0), 7.7(4)
α, β, γ (°)	90, 90, 90	90, 90, 90
V (Å^3)	5556.0	2790.5
ρ (g/cm^3)	1.72	2.29
F(000)	2880	1440
	**Data collection**	
Temperature (K)	293	293
Radiation (Å)	electrons, 0.02508	electrons, 0.02508
Number of crystals	7	2
d_{min}, d_{max} (Å)	0.76, 8.77	0.81, 6.25
Dataset (h, k, l)	-25→25, -23→24, -16→15	-22→22, -22→24, -8→8
Tot., Uniq. data, R_{int}	20597, 4488, 0.208	1941, 904, 0.101
Observed Data [$I > 2.0\sigma(I)$]	3029	555
	**Refinement**	
$N_{reflections}$, $N_{parameters}$, $N_{restraints}$	4488, 332, 0	904, 44, 0
$R1$ [$F^2 > 2.0\sigma(F^2)$], $wR2$, S	0.1960, 0.4735, 1.08	0.2910, 0.6472, 2.16
Δ/σ (max., avg.)	0.06 (EXTI), 0.00	0.00, 0.00
ρ_{min}, ρ_{max} (V/Å^3)	-0.28, 0.28	-0.24, 0.40

Table S6 Crystallographic details for the two clustered data sets of PST-20 & ZSM-25 mixed phase sample.

**Crystal data**		
Sample	PST-20 (mixed phase)	ZSM-25 (mixed phase)
Formula	$[Si_{2640}O_{5280}]$	$[Si_{1440}O_{2880}]$
Formula Weight (g/mol)	158637.6	86529.6
Crystal System	cubic	cubic
Space group	$Im\bar{3}m$ (No. 229)	$Im\bar{3}m$ (No. 229)
a, b, c (Å)	55.07, 55.07, 55.07	45.07, 45.07, 45.07
α, β, γ (°)	90, 90, 90	90, 90, 90
V (Å^3)	167011.1	91550.9
ρ (g/cm^3)	1.577	1.569
F(000)	79200	43200
**Data collection**		
Temperature (K)	293	293
Radiation (Å)	electrons, 0.02508	electrons, 0.02508
Number of crystals	7	2
d_{min}, d_{max} (Å)	1.46, 17.54	1.46, 18.44
Dataset (h, k, l)	-37→37, -37→37, -37→37	-30→30, -27→27, -29→29
Tot., Uniq. data, R_{int}	92346, 2647, 0.380	15974, 1492, 0.332
Observed Data [$I > 2.0\sigma(I)$]	1179	695
**Refinement**		
$N_{reflections}$, $N_{parameters}$, $N_{restraints}$	2647, 267, 290	1492, 150, 160
$R1$ [$F^2 > 2.0\sigma(F^2)$], $wR2$, S	0.4900, 0.8583, 3.22	0.4863, 0.8448, 3.07
Δ/σ (max., avg.)	0.00, 0.00	0.00, 0.00
ρ_{min}, ρ_{max} (V/Å^3)	-0.42, 0.35	-0.25, 0.25

Table S7 Comparison of data statistics from refinements of PCN-416 structure against manual cRED and merged SerialRED data.

	Manual cRED[1]	SerialRED
space group	$I\bar{4}2m$	$I\bar{4}2m$
Unit cell parameter	a = 16.7 Å, c = 30.1 Å	a = 16.5 Å, c = 29.8 Å
Number of merged data sets	1	12
Resolution	1.05	0.90
Completeness	100.0	96.8
No. Unique reflections	1918	2825
No. Observed reflections	912	1254
*R*1, *R*1(all)	0.258, 0.317	0.216, 0.278
Goof	1.50	1.30

[1] From Yuan *et al.*, 2018.

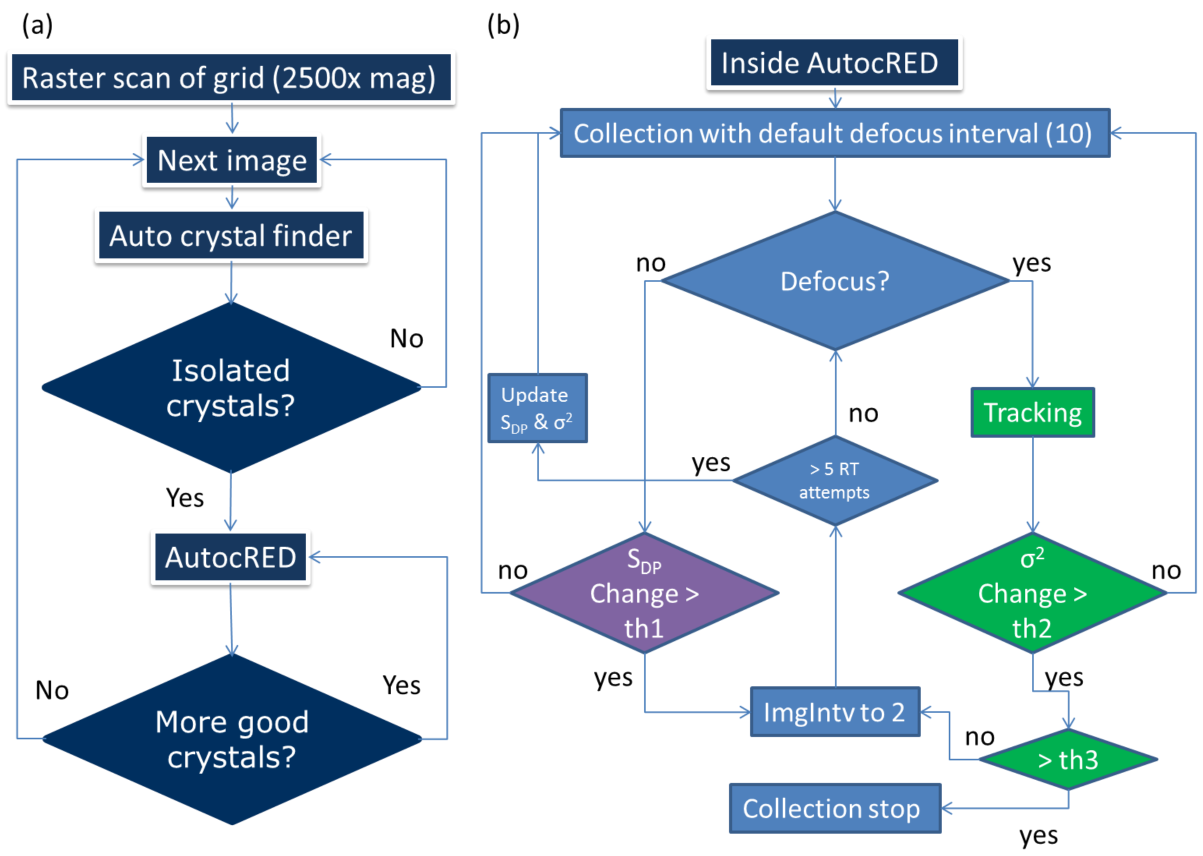

Figure S1 Flowcharts for the multi-crystal data acquisition. (a) Automatic crystal finding in a serial manner. If a suitable, isolated crystal is found, the function for cRED data collection is activated. (b) Automated cRED data collection procedure. Steps performed in focused diffraction condition are shown in purple, while those in defocused condition are shown in green. In the focused condition, the image scale (S_{DP}) is checked, and in the defocused condition, the image variance ($\sigma2_{defocus}$) is checked. If the change (see Eq. s3 & s4) in either parameter is larger than the threshold value, robust tracking (abbreviated as RT in the figure) is activated by setting the defocus frequency to 2. Th1, th2, and th3 are the three threshold numbers set for deciding whether the crystal has moved (partially) out of the electron beam. Typical numbers used are: th1 = 0.15, th2 = 0.5 and th3 = 0.8.

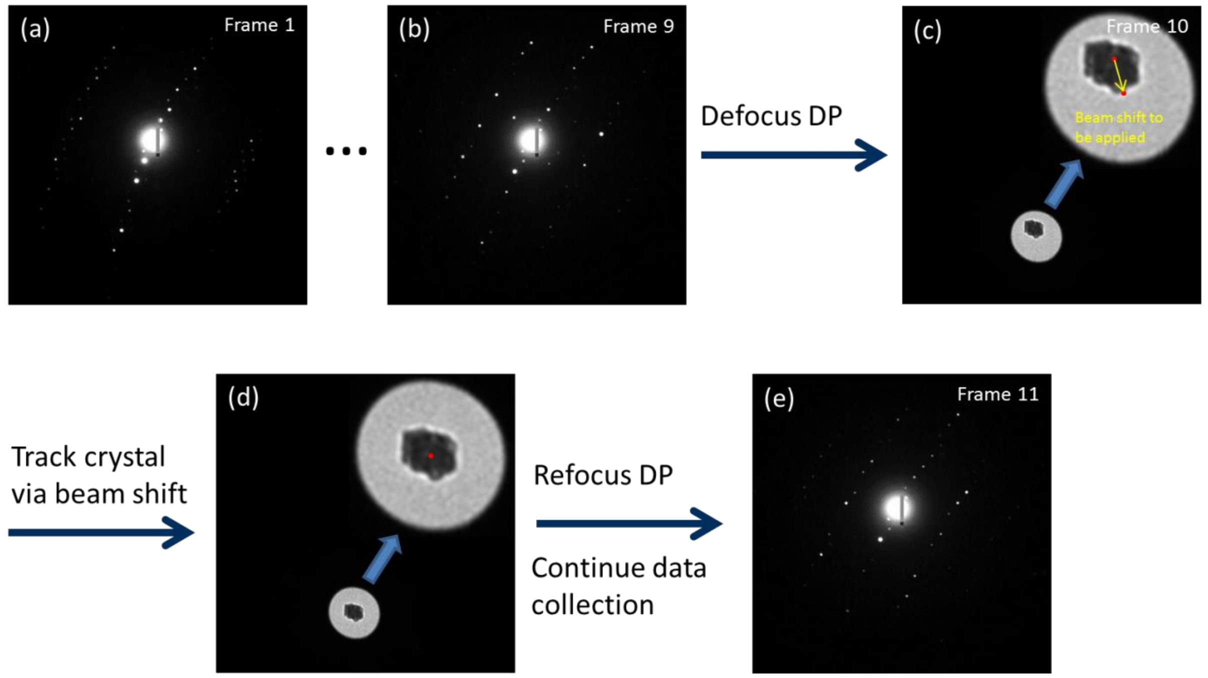

Figure S2 Schematic illustration of the tracking algorithm. Diffraction patterns are defocused at regular intervals (i.e. every 10th pattern) to observe the position of the crystal. (a-b) Diffraction patterns. (c) The defocused pattern. Relative offset of the crystal position from the center of the probe is estimated and (d) the position of the probe is adjusted accordingly by changing the beam shift deflectors. After applying beam shift, the image shift deflectors are used for aligning the diffraction pattern. (e) Continued diffraction data collection.

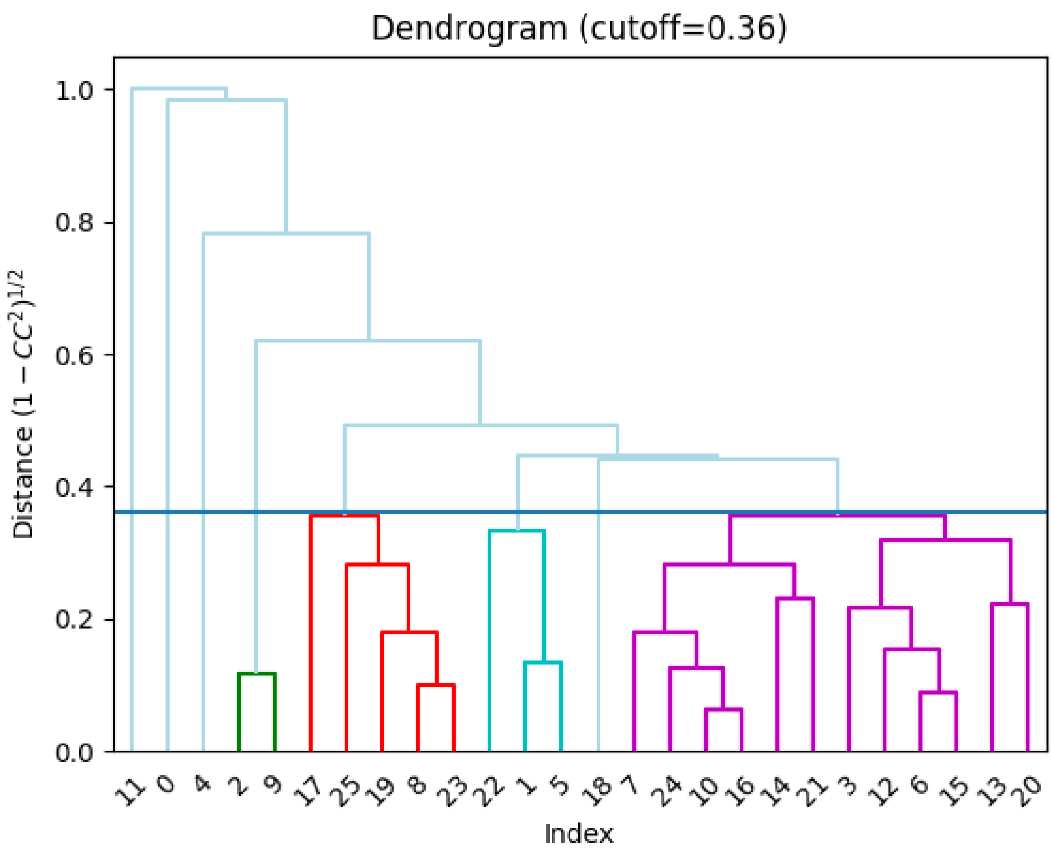

Figure S3 Reflection-based clustering for PCN-416 experiment. The magenta cluster, consisting of 12 single data sets, possessed the best data statistics in terms of data completeness and I/σ value, and thus was used for structure determination and refinement.

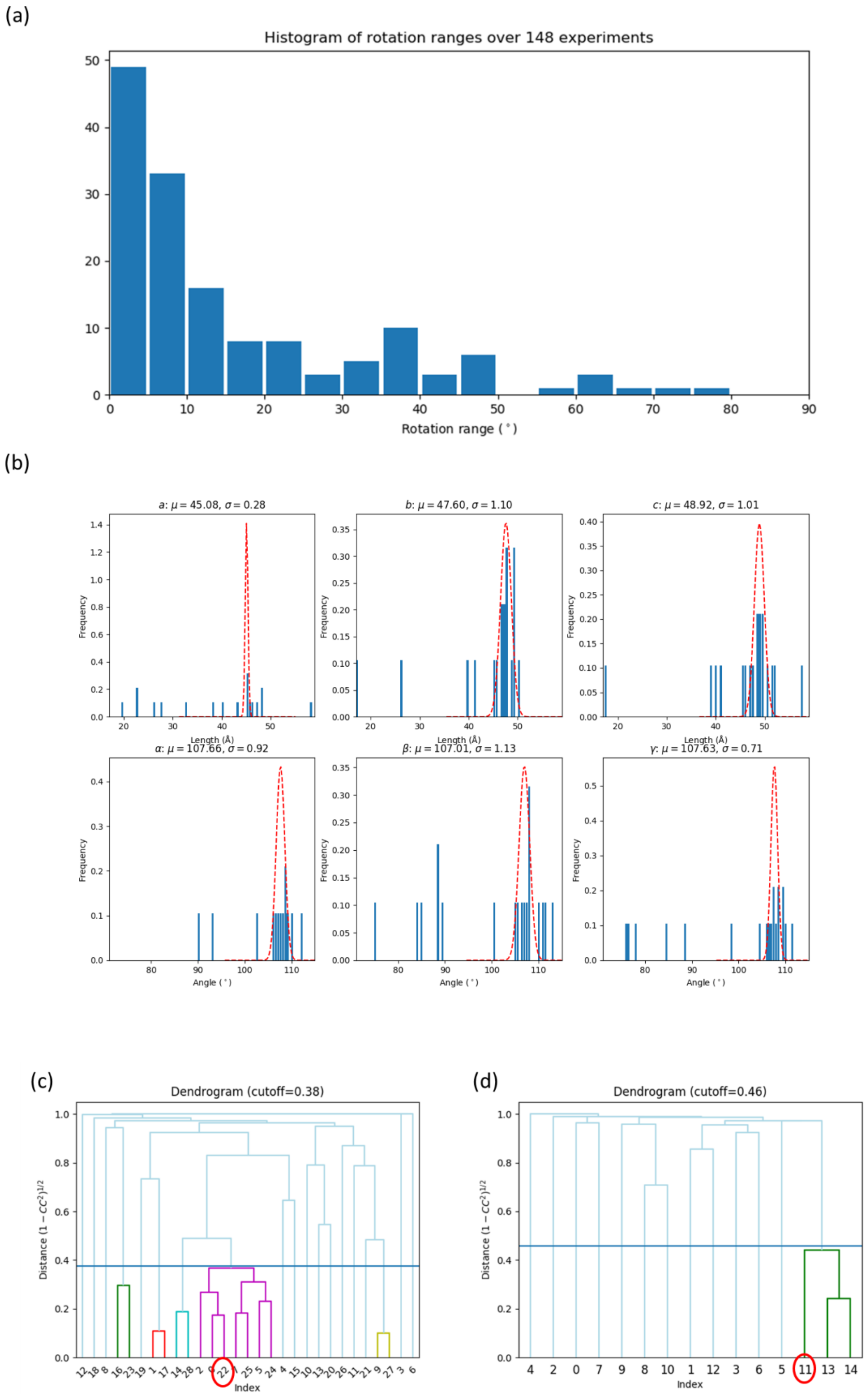
(a)
Histogram of rotation ranges over 148 experiments
Rotation range (°)
(b)
a: μ = 45.08, σ = 0.28
b: μ = 47.60, σ = 1.10
c: μ = 48.92, σ = 1.01
Frequency
Length (Å)
α: μ = 107.66, σ = 0.92
β: μ = 107.01, σ = 1.13
γ: μ = 107.63, σ = 0.71
Angle (°)
(c)
Dendrogram (cutoff=0.38)
Distance (1 − CC²)^1/2
Index
(d)
Dendrogram (cutoff=0.46)
4 2 0 7 9 8 10 1 12 3 6 5 11 13 14

Figure S4 (a) Histogram of the PST-20 experiment. This also shows what a typical auto data collection looks like. The majority of the experiments terminate at a very early stage (i.e. < 10°) for reasons such as a sudden movement at the beginning of the rotation, the *Z* height being too far from the eucentric condition, the crystal appearing to be an agglomerate, etc. (b) Unit cell parameter histograms from the initial XDS step. The average unit cell parameters are: *a*=45.08(28) Å, *b*=47.60(1.10) Å, *c*=48.92(1.01) Å, α=107.66(92)°, *β*=107.01(1.13)°, *γ*=107.63(71)°. By looking at the distribution of the lengths, one may conclude that the sample consists of at least ZSM-25 and PST-20. (c-d) The cutoff of CC(I) value for (c)PST-20 and (d) ZSM-25. An interesting phenomenon is that, among all the indexed data sets, 7 data sets could be successfully indexed by both unit cells. This can be due to the fact that we left the indexing successfully rate to be default (25%) in XDS. By looking at (d), a lot of the data sets are actually far from each other; when we made the cutoff of CC(I)'s to be 92.5% and 88.8% for PST-20 and ZSM-25 as shown in the figures, the circled data sets (No. 22 in b and No. 11 in c) are actually the same data set. We had to decide ourselves that since this data seemed to be more correlated to PST-20 from the dendrogram, it is assigned as a PST-20 data set. Finally ZSM-25 was cut at a lower distance to exclude dataset #11.

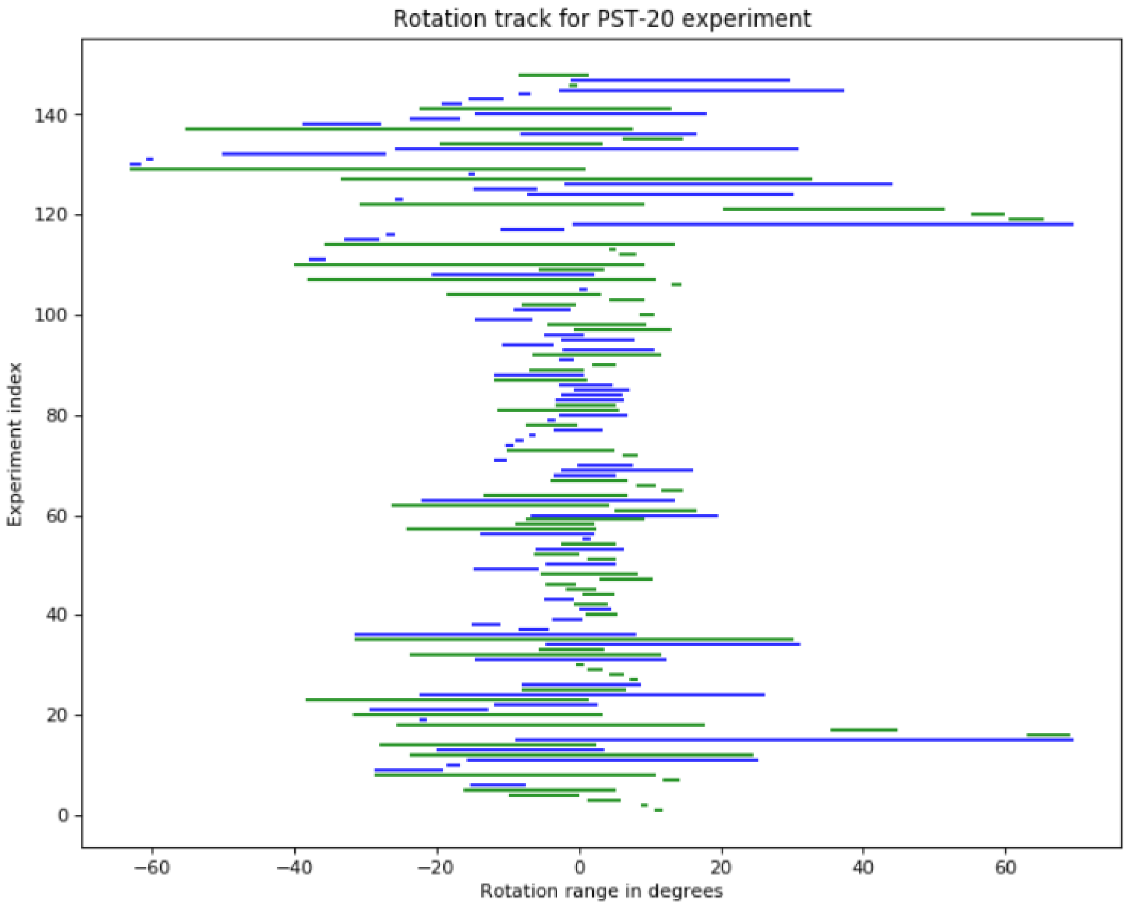

Figure S5 Rotation traces for the auto-run PST-20 experiment. The *y* axis ("experimental index") marks the chronological order for the 148 experiments (starting from index 1). The blue lines indicate that the rotation was towards positive direction, and the green lines negative direction. The method takes a strategy of starting rotation from wherever it currently is, targeting either +70° or -70° whichever is further away from the initial angle readout, and stopping rotation when the crystal is detected to be out of the beam. This strategy is of course not an optimal approach for an overall high rotation range purpose. However, considering that the rotation speed is global, it is simply inefficient to wait for the goniometer tilt to reach high angles every time before starting a rotation experiment, which could be more beneficial for achieving higher rotation ranges overall. At every position, a rotation of 0.5° is always done in order to eliminate possible backlash in the tilt direction before checking if there is any suitable crystal and calculating the crystal positions. This explains the discontinuities in the figure.
